# Recent Investigations on Thiocyanate-Free Ruthenium(II) 2,2′-Bipyridyl Complexes for Dye-Sensitized Solar Cells

**DOI:** 10.3390/molecules26247638

**Published:** 2021-12-16

**Authors:** Luca Mauri, Alessia Colombo, Claudia Dragonetti, Dominique Roberto, Francesco Fagnani

**Affiliations:** Department of Chemistry, University of Milan, UdR-INSTM, Via C. Golgi 19, I-20133 Milan, Italy; luca.mauri@unimi.it (L.M.); alessia.colombo@unimi.it (A.C.); claudia.dragonetti@unimi.it (C.D.); dominique.roberto@unimi.it (D.R.)

**Keywords:** dye-sensitized solar cells, photosensitizers, thiocyanate-free ruthenium dyes, bipyridine ruthenium complexes

## Abstract

Three decades ago, dye-sensitized solar cells (DSSCs) emerged as a method for harnessing the energy of the sun and for converting it into electricity. Since then, a lot of work has been devoted to create better global photovoltaic efficiencies and long term stability. Among photosensitizers for DSSCs, thiocyanate-free ruthenium(II) complexes have gained increasing interest due to their better stability compared to conventional thiocyanate-based complexes, such as benchmark dyes N719 and Z907. In this mini-review, two classes of thiocyanate-free Ru(II) complexes are presented: (a) bis-bipyridyl compounds bearing an ancillary cyclometalating bidentate ligand; (b) bipyridyl compounds bearing non-cyclometalating ancillary ligands. The coverage, mainly from 2014 up to now, is not exhaustive, but illustrates the most recent design strategies and photovoltaic properties of these two families of ruthenium(II) dyes.

## 1. Introduction

Among the different types of photovoltaic devices developed in recent decades, dye-sensitized solar cells (DSSCs) have attracted growing interest, reaching remarkable improvements of their performances. This has been possible since all the components of such cells have been deeply studied, trying to optimize the sensitizer, the electrolytic mixture and the fabrication method.

The first examples of these solar cells were fabricated, tested and published by Grätzel and O’Regan in 1991 [1], reaching, in the following years, record efficiencies of 12% [2].

A DSSC is generally based on a dye molecule (a purely organic or an organometallic compound) adsorbed on a semiconductor layer, such as titanium dioxide, deposited on a photoanode made of a conductive oxide, such as fluoride-doped tin oxide (FTO). Between the anode and the cathode, the filling of the device is composed by an electrolytic mixture of a redox couple (more commonly the I^−^/I_3_^−^ one, but recently an important series of earth-abundant metal complexes has been tested [3,4,5,6,7,8,9,10] together with some organic or inorganic additives [11]).

When the cell is irradiated by sunlight, the dye molecule is excited to a higher energy level, from which it loses an electron; the electron is injected into the titania layer, reaches the photoanode and then arrives at the cathode by means of an external circuit.

Thanks to a catalyst (e.g., platinum or graphite) present on the cathode, the electron recombines with the oxidized species of the redox couple, forming the reduced one. The reduced species then reacts with the cationic form of the dye, regenerating it and completing the cycle.

**Figure 1:**
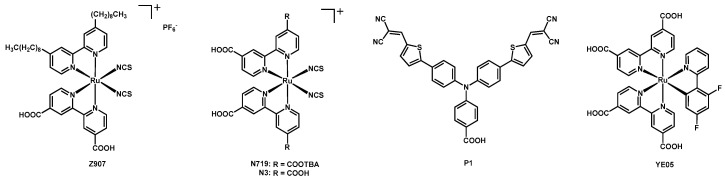
Structure of the reference dyes **Z907**, **N719**, **N3**, **P1,** and **YE05**.

Up to now, one of the most used and best-performing dyes is the Ru(II) complex **N719**, having two ancillary NCS ligands and two anchoring 2,2′-bipyridine-4,4′-dicarboxylic acid (dcbipy) ligands, in which two of the four carboxylic groups are present as tetrabutylammonium (TBA) salts (structure in Figure 1).

One of the main problems coming from the presence of the thiocyanate ancillary ligand is its ambidentate nature, since it can coordinate to the metal through the nitrogen or the sulfur atom; moreover, it is quite labile and can be easily replaced by other species contained in the device, such as 4-*tert*-butylpyridine (TBP) often included as additive in the redox mixture.

To overcome the mentioned problems with thiocyanates, many research groups have investigated new bidentate or polydentate chelating ligands to replace the NCS ligands.

This review aims to illustrate some of the papers published in the last seven years in the field of NCS-free bipyridine ruthenium(II) complexes employed as dyes in DSSCs, as an update of the review of Dragonetti, Abbotto et al. of 2014 [12]. It is worth mentioning that other important reviews on this topic have been published in the last few years, providing a wide overview on the progresses made in the field of the dye design and synthesis, presenting both metal-based sensitizers, purely organic compounds, and natural derivatives [13,14,15].

A good laboratory practice is to report the photoelectrochemical performance of the DSSC employing the dye under investigation with that of a control cell with **N719** as standard dye, in order to allow for an easier comparison of results obtained for cells fabricated in different laboratories. Therefore, when possible, we report in the Tables the photoconversion efficiencies of devices based on the investigated photosensitizer relative to **N719** set at 100% (η_rel_); this comparison has been made only in the case of devices fabricated with the exact same composition and formulation of both the dye and the electrolyte solution. When two **N719**-based devices, prepared in the same conditions, were presented (Table 1, entries 20–21), we considered the average value of the efficiency.

## 2. Ruthenium Complexes with Bipyridine Ligands as Sensitizers in DSSCs

When considering ruthenium complexes as dyes for DSSC applications, 2,2′-bipyridine (generally abbreviated as bipy) is certainly one of the most employed chelating ligands for solar light harvesting; its functionalization with carboxylic or phosphonic groups allows the anchoring of the dye on the semiconductor surface.

### 2.1. Ruthenium Compounds with Cyclometalating Ligands

One of the first to realize the usefulness of cyclometalating 2-phenylpyridines as ligands in DSSC dyes was Berlinguette, with many important works in this field [21,22,23,24,25].

More recently, a wide range of this type of ligands have been obtained and tested in Ru-based sensitizers, providing interesting results.

**Figure 2:**
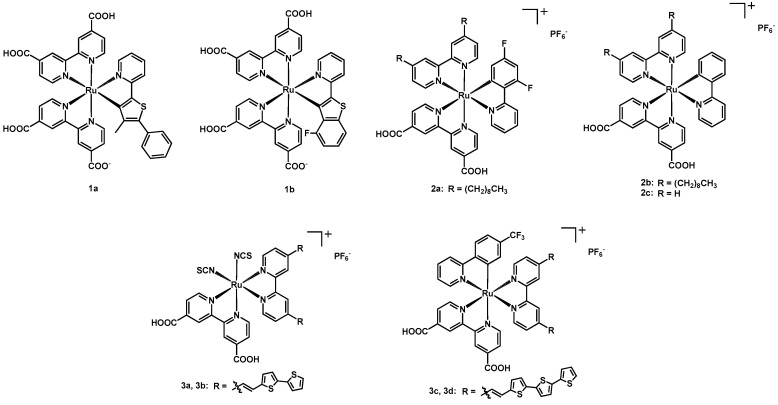
Structure of dyes **1a**–**1b**, **2a**–**2c**, and **3a**–**3d**.

In 2014, two thiophene-incorporated ruthenium complexes (**1a** and **1b**, structure in Figure 2) were published by Li, Su, Wang et al. [16]. These dyes had two dcbipy ligands and a cyclometalated N^C ligand based on 2-thienylpyridine. In particular, complex **1a** had a 2-(4-methyl-5-phenyl-thiophen-2-yl)pyridine ligand, whereas **1b** a 2-(4-fluorobenzo[b]thiophene-2-yl)pyridine.

These dyes showed broader absorption spectra and higher molar extinction coefficients if compared to the reference sensitizer **N719** when absorbed on TiO_2_ layers. The replacement of the two thiocyanate ligands with the cyclometalating 2-thienylpyridines provided slightly higher HOMO and LUMO levels (0.01~0.03 eV), but a comparable HOMO-LUMO gap (~1.60 eV), hence both **1a** and **1b** were suitable sensitizers for DSSCs.

The photovoltaic performances were tested working with the I^-^/I_3_^-^ redox couple under AM 1.5G conditions (100 mW cm^−2^). The performance data are listed in Table 1 (entries 1–2): with an efficiency of 4.22%, **1b** was the most promising dye, but not yet able to compete with the standard dye **N719** (η = 9.37%, entry 3). Additionally, the IPCE values followed the trend **N719** > **1b** > **1a**. In order to explain these results, the electrochemical impedance spectroscopy (EIS) technique was used; with respect to **N719**, compounds **1a** and **1b** gave lower electron-transport resistances and higher electrochemical impedances, indicating a more efficient electron recombination of TiO_2_ electrons with acceptor species in the electrolyte. This was also supported by the shorter electron lifetimes and the lower electron effective diffusion coefficients: 17.8 ms and 2.36 × 10^−4^ cm^2^/s, respectively, for **1a**, 31.6 ms and 2.69 × 10^−4^ cm^2^/s for **1b**, 121.2 ms and 3.90 × 10^−4^ cm^2^/s for **N719**.

In the same year, Hamann and coworkers published the two new Ru(II) complexes **2a** and **2b** (Figure 2) [17], having two bipy ligands and a cyclometalating 2-phenylpyridine. Both complexes had a dcbipy and a 4,4′-dinonyl-2,2′-bipyridine (dnbipy); the aim of the nonyl chains was to provide a sufficient steric hindrance to avoid the ease of access of the redox mediators to the titania surface. In **2a,** the third ligand was a 2-(2,4-difluorophenyl)pyridine, whereas in **2b** a simple 2-phenylpyridine. The properties of these new compounds were compared with those of the previously known sensitizers **2c** (the same structure as **2b** but without the nonyl chains) and **Z907** (see Figure 1: same bipyridines of the new complexes but with two NCS ligands instead of the phenylpyridine).

All ruthenium complexes were tested working with [Co(dmbipy)_3_]^2+/3+^ as redox couple (dmbipy = 4,4′-dimethyl-2,2′-bipyridine) and under AM 1.5G conditions (100 mW cm^−2^). Compound **2b** presented very similar results with respect to the reference **2c**, despite the introduction of the nonyl chains on the bipy ligand; better values were obtained with **2a**, whose J_sc_ value was remarkably higher than that of **2b** (3.26 vs. 1.96 mA cm^−2^; Table 1, entries 4 and 6). However, the best data were still those of the benchmark dye **Z907**, which showed an efficiency of 1.33% and a photocurrent of 4.86 mA cm^−2^ (entry 7).

In order to further hamper the recombination of the oxidized redox species, other devices were produced by adding an ultra-thin layer of alumina on the TiO_2_, by means of atomic layer deposition (ALD) technique; the presence of the additional coating was beneficial to the measured values, with an important increase in all parameters. Additionally, in this case, **Z907** was the best performing sensitizer (entry 11), followed by **2a**, **2b**, and **2c**, in the same order as shown before in the absence of the alumina layer (entries 8–11).

In the same year Islam, El-Shafei, and coworkers presented four new sensitizers having a dcbipy and a bithiophene- or terthiophene-based bipyridinic ligand [18]. The remaining coordination centers were occupied by two NCS ligands in the case of **3a** and **3b**, and by a 2-(4-trifluoromethylphenyl)pyridine in **3c** and **3d** (Figure 2). The substitution of the thiocyanates with a cyclometalated ligand provided a smaller HOMO-LUMO gap (resulting in a red shift of the absorption), while the addition of a third thiophene ring in the light-harvesting bipyridine ligand increased the extinction coefficient of the sensitizers, giving the possibility of a lower dye loading and thinner TiO_2_ layers in the devices.

The photovoltaic properties of the four complexes were compared with those of the classical **N719** dye, also testing the effect of different amounts of TBP as an additive in the electrolytic mixture. The most promising compound was the NCS-based **3b**, with a J_sc_ of 20.585 mA cm^−2^, a V_oc_ of 0.660 V and a conversion efficiency of 9.71% (Table 1, entry 14). Despite the lower efficiency (8.83 vs. 9.12%), also the photocurrent density of **3a** was higher than that of **N719** (20.454 vs. 17.160 mA cm^−2^; entries 12 and 20); in this case, such results could be explained considering a poorer charge separation and/or higher recombination reactions at the dye/titania/electrolyte interface. For all the parameters, dyes **3c** and **3d** registered worse results (entries 16–19), due to a slower dye regeneration (electron lifetimes of 13 and 18 μs, respectively, much longer than the 6 and 4 μs obtained with **3a** and **3b**) and a lower driving force, under the minimum required threshold of 0.25 eV. Table 1 resumes data for the mentioned solar cells.

**Figure 3:**
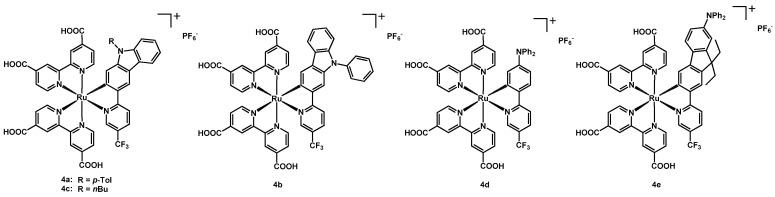
Structure of dyes **4a**–**4e**.

Other five Ru(II) complexes were synthesized and published in the year 2014 by Ho, Chen, Wong et al. [19]. These cationic dyes had two dcbipy and a cyclometalated N^C ligand with the base structure of a 2-phenylpyridine with a CF_3_ group in position 5. Compounds **4a**, **4b**, and **4c** had an N-substituted pyridyl-carbazole, where the substituents were a *p*-tolyl, a phenyl and a *n*-butyl, respectively; **4d** presented a diphenylamino group on the phenylpyridine ligand, while **4e** had a diphenylamino-pyridyl-fluorene scaffold. The structure of the dyes is shown in Figure 3.

These sensitizers showed a higher extinction coefficient when compared to the standard **N719** dye and, as already mentioned, this could allow for a thinner layer of titania in the DSSC and for a lower possibility of recombination between the species. All data are presented in Table 1. The highest solar conversion was obtained by **4a**, that reached a maximum IPCE value of 56% at 560 nm; the same dye also achieved the best J_sc_ (8.06 mA cm^−2^, Table 1, entry 22) and η (3.39%), much higher than that of the other four complexes (entries 23–26). It can be observed that these performances came from the use of carbazole as electron-donating moiety in the N^C ligand, the best performance being reached with the *p*-tolyl group on the nitrogen atom.

**Figure 4:**
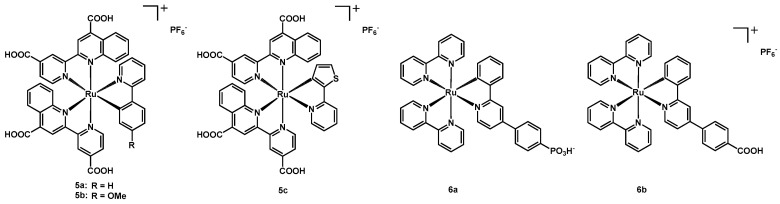
Structure of dyes **5a**–**5c** and **6a**–**6b**.

Up to now all the presented works had COOH-substituted bipyridines as anchoring ligands, while in the paper of Funaki, Sugihara and coworkers from 2014 [20] two phenyl-quinolines were employed. The cyclometalating ligands of these compounds were a simple 2-phenylpyridine for **5a**, a 2-(4-methoxyphenyl)pyridine for **5b** and a 2-(2-thienyl)pyridine for **5c**. In all cases the complex was cationic and had a PF_6_^−^ counteranion (see Figure 4 for the structure of the compounds).

In order to have a proper adsorption of the dye onto the TiO_2_, the presence of deoxycholic acid as co-adsorbent was necessary; moreover, different sensitizer concentrations were tested and the measurements were carried out under AM 1.5 irradiation (100 mW cm^−2^), employing two different electrolytic solutions in acetonitrile: the former containing 0.6 M 1,2-dimethyl-3-propylimidazolium iodide + 0.05 M I_2_ + 0.1 M LiI, the latter having 0.05 M I_2_ + 2.0 M LiI; also the benchmark dye **N719** was used as a reference. Compound **5b** gave the best results for all parameters, originating a J_sc_ of 15.24 mA cm^−2^, a V_oc_ of 468 mV and an efficiency of 4.64% (Table 1, entry 28) when the first electrolytic solution was used; this efficiency largely overcame the values of both **5a** and **5c** (entries 27 and 29), due to higher photocurrent and voltage. Table 1 lists all photovoltaic data.

A dramatic improvement of the measured parameters was achieved with the second solution, containing a much larger amount of LiI, which was thought to positively shift the conduction-band edge of the titania. When compared to **N719**, **5b** showed a better photo-response (entry 32) in the longer wavelengths, reaching an IPCE of 76% and having a panchromatic sensitization not only in the visible region but also in the NIR, up to 1020 nm. As stated by the authors, this was the best result for a cyclometalated Ru(II) complex in the NIR region. This red-shift was due to the use of a strong electron-donating N^C ligand and of a more π-extended anchoring ligand.

In 2016, Housecroft and coworkers published a paper in which two derivatives of complex [Ru(bipy)_2_(Cl-ppy)][PF_6_] (Cl-ppy = 4-chloro-2-phenylpyridine) were explored [26]. In the first compound (**6a**) the N^C ligand was expanded through a 4-PO_3_H-phenyl moiety to form a zwitterionic dye of Ru^2+^, while in the second (**6b**) via a 4-COOH-phenyl ring (Figure 4). These phosphonic and carboxylic substituents aimed at the introduction of anchoring groups to link the sensitizers to the semiconductor, since the other ligands on the metal were simple bipyridines and not dcbipy as often presented before.

Then, **6a** was tested in DSSCs together with the organic reference sensitizer **P1** (depicted in Figure 1), adsorbed on one or two layers of NiO. Independently on the number of NiO layers, the photovoltaic performances of **6a** were better than those of the standard **P1** dye, reaching a J_sc_ of 2.18 mA cm^−2^ in the case of a single layer and of 3.38 mA cm^−2^ in the presence of two layers (Table 2, entries 1–8). The efficiency of these devices had a maximum value of 0.116% (entry 7).

**Figure 5:**
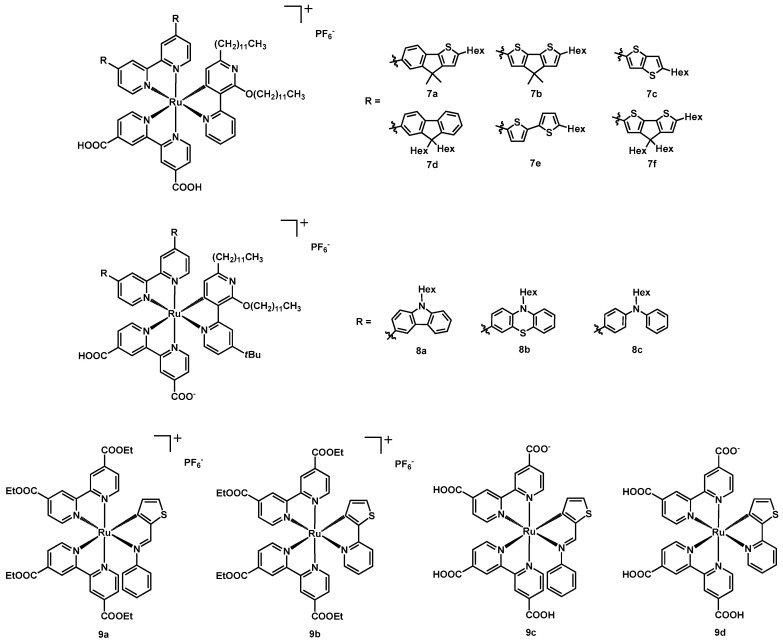
Structure of dyes **7a**–**7f**, **8a**–**8c**, and **9a**–**9d**.

In 2016, Nazeeruddin et al. developed a series of new tris-heteroleptic cyclometalated Ru(II) complexes [27] having a dcbipy molecule as anchoring ligand, a bipyridine with aromatic substituents in the 4 and 4′ positions as main light-harvesting moiety, and a 2,6-didodecyloxy-3,2′-bipyridine as ancillary C^N ligand; the long alkyl chains prevented the access of the redox mediators to the semiconductor surface. The structures of the six new dyes **7a**–**7f** and of the investigated substituents are shown in Figure 5.

In order to test the performances of these sensitizers, some DSSCs were produced, employing [Co(Phen)_3_]^2+/3+^ (Phen = 1,10-phenanthroline) as redox couple. Surprisingly, the best results came from compound **7c** (Table 2, entry 11), with a short-circuit photocurrent of 14.55 mA cm^−2^ and a η of 9.4% under AM 1.5 conditions and a power of 100 mW cm^−2^; this was not attended because of the limited absorption range of this dye, but desorption studies showed that the amount of sensitizer loaded onto the titania layer was much higher with respect to the other five compounds. Additionally, **6f** provided a large loading, but its inefficiency in the dye regeneration made it produce a lower IPCE. It can be concluded that the higher dye loading of **7c** was responsible for an increased J_sc_, hence for a higher efficiency, despite its low extinction coefficient and its narrow absorption range. Table 2 contains all data for the discussed sensitizers.

Another useful take-home message is that the proper compromise between the various parameters is crucial to achieve the best cell performance, even if it is not easy to take into account all the factors which play a role in the complex processes occurring in a DSSC.

Four of the aforementioned ruthenium(II) sensitizers (**7b**, **7c**, **7d**, and **7f**) were published by the same group and in the same year in another paper [28], this time focusing the attention on the role of sulfur atoms in the aromatic substituents of the light-harvesting bipyridine. These moieties were different in the number of the thiophene rings and in the way they were connected: **7d** had no sulfur but a fluorene-based group, **6c** presented a thienothiophene, while **7b** and **7f** a cyclopentadieno-dithiophene unit with methyl or hexyl chains. These ligands were designed in order to prove if the presence of sulfur in the dye structure had a positive or negative effect on the cell properties when the classical I^−^/I_3_^−^ redox couple is chosen for the electrolytic solution.

Differently from the previous work, this time the best results were given by sensitizer **7d**, (Table 2, entry 23), with the highest V_oc_ (0.694 V) and the best efficiency (7.2%, under 1 Sun simulated conditions), even if with the lowest J_sc_ (13.84 mA cm^−2^). Complete data of the produced solar cells can be found in Table 2. Based on these data and on the in-depth absorption, electrochemical, and theoretical studies made by the authors, it can be concluded that the presence of sulfur in iodine-containing DSSCs is detrimental for the cell performances, due to the interaction between sulfur- and iodine-based species. In fact, sulfur-free dye **7d** was characterized by an increased electron recombination efficiency, despite the lower dye regeneration yield.

In 2017, Aghazada, Ren, Wang et al. [29] described three tris-heteroleptic cyclometalated Ru(II) complexes (**8a**–**8c**) bearing different donor groups on the bipyridinic ligand. The complexes were based on a dcbipy anchoring ligand, a 4-(tert-butyl)-2’,6’-bis(dodecyloxy)-2,3’-bipyridine N^C ligand, and a 4,4′-substituted bipy. The structure of the mentioned compounds are shown in Figure 5.

Each dye had a high molar absorption coefficient almost over the entire visible spectrum. For what concerned the electrochemical properties, the three sensitizers showed two oxidations; the first one was attributed to the Ru^3+/2+^ redox reaction, the second one to the oxidation of the donor groups on the ancillary ligands; while the first oxidation was found to be completely reversible, the second one was not. However, the authors observed that only the first oxidation occurred in a DSSC; since the dye was present in a reducing environment, the second oxidation could not take place and did not represent an issue for the performances of the photovoltaic device.

Moving to the photovoltaic performances, the synthesized dyes were tested using the redox couple [Co(Phen)_3_]^3+/2+^ under AM 1.5G irradiation. Except for the FF, the order of the V_OC_, the J_SC_ and the efficiency was the following: **8b** > **8a** > **8c**, Table 2 entries 25–27.

In the same year, four new cycloruthenated complexes (**9a**, **9b**, **9c,** and **9d**, structure in Figure 5) containing ortho-metalated thiophenes were published by Medved’ko, Ivanov et al. [27]. The complexes were based on two dcbipy, which carboxyl groups were present as ethyl esters in compounds **9a** and **9b**, while they were free carboxyl groups in compounds **9c** and **9d**. Complexes **9a** and **9c** had a N-(thiophen-2-ylmethyliden)aniline ligand, whereas complexes **9b** and **9d** had a 2-(thiophen-2-yl)pyridine.

All dyes showed intense and broad absorption bands with high molar absorption coefficients, and absorption spectra almost over the entire visible spectrum. All the complexes were found to be redox-active with completely reversible oxidations.

The photovoltaic performances of DSSCs fabricated with the dyes **9c** and **9d** were tested using the method of photopotential and photocurrent transient to study the dye regeneration process, under AM 1.5G illumination conditions and compared to those of the reference dye **N3** (Figure 1) and of the dyes **1a** and **1b** previously published by Li et al. [16]. The dye **9c** showed a J_SC_ and a V_OC_ that exceed those of dye **9d**, but not those of the standard dye. The same trend was observed for the efficiency. However, the dyes **9c** and **9d** represented an improvement respect to the previously published dyes **1a** and **1b** both in terms of J_SC_, V_OC_ and efficiency. The experimental results are listed in Table 2 (entries 28–30).

**Figure 6:**
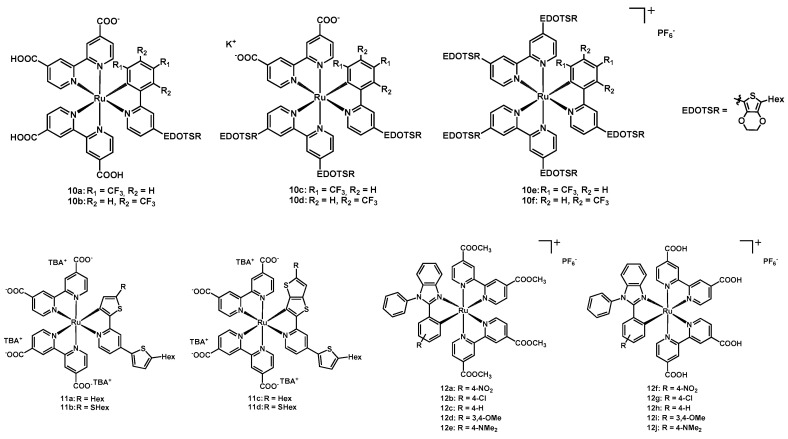
Structure of dyes **10a**–**10f**, **11a**–**11d**, and **12a**–**12j**.

A further example of an interesting family of NCS-free ruthenium(II) dyes was published in 2017 by Wu’s group [31]. The new complexes shared a cyclometalating 2-phenyl-pyridine with a different location of CF_3_ substituents, and two anchoring bipy ligands with a different number of carboxylic and/or EDOTSR moieties (where EDOTSR stands for 5-hexylthio-3,4-ethylendioxythiophen-2-yl, whose structure is shown in Figure 6).

In compounds **10a**, **10c,** and **10e** the trifluoromethyl groups were in the *ortho* and *para* positions with respect to the Ru-bound carbon atom of the phenyl ring, while in the case of **10b**, **10d,** and **10f** they were in the *meta* positions. Additionally, **10a** and **10b** contained two dcbipy ligands, while **10c** and **10d** had one dcbipy and one EDOTSR-bearing bipy; finally, **10e** and **10f** had two EDOTSR-substituted bipy.

This work aimed at understanding how the photovoltaic properties of the mentioned Ru^2+^ dyes were influenced by the presence and position of the electron-withdrawing CF_3_ groups on the N^C ligand. It was observed that the *meta*-substitution reduced the electron density on the metal less than that in the *ortho* and *para* positions, and also provided a better interaction between the I^-^ and the oxidized sensitizer, due to less steric hindrance. As a result, the best efficiency value (9.03%) was achieved by **10b** (Table 2, entry 32) which also had the highest J_sc_ of 18.40 mA cm^−2^; these performances overcame those of the devices produced with the classical **N719** dye (entry 37), in the same fabrication conditions. Table 2 lists all detailed data for these dyes.

Another paper with four new dyes was published in 2018 by the same group [32], this time choosing as only anchoring ligands the tetrabutylammonium salts of dcbipy and as N^C ligand a thienyl- or thienothienyl-pyridine, bearing a 5-Hex-thienyl moiety in the 4 position of the pyridine and different alkyl chains on the cyclometalating ring. Figure 6 shows the structures of the novel compounds **11a**–**11d**, through which the authors explored the effect of having a simple thiophene or a thienothiophene on the pyridine, and also the influence of the presence of a sulfur atom between the ring and the hexyl chain.

As expected, the highest dye loading onto the titania layer was achieved by complexes **11a** and **11b** (8.76 × 10^−8^ and 14.5 × 10^−8^ mol cm^−2^, respectively), being the less sterically hindered molecules.

The short-circuit current was in the range 14.9 to 16.5 mA cm^−2^ (Table 2, entries 38–41), in all cases higher than the value achieved using **N719** (entry 42) in reference DSSCs; on the contrary, this standard dye still had the best V_oc_ (0.67 V), FF (0.73) and η (7.26%). Moreover, the new dyes showed an open-circuit voltage, Fill Factor and efficiency very close to each other. Data are presented in Table 2.

As stated by the authors, an important feature of sulfur-containing sensitizers was represented by the positive interaction between the soft sulfur atom and the soft iodide anion, producing a good dye regeneration.

In 2020, Lavrova, Mishurinskiy et al. [33] published five new ruthenium-based dyes having two anchoring dcmbipy ligands and a *N*-phenyl-2-arylbenzimidazole cyclometalating ligand bearing different substituents (complexes **12a**–**12e**; Figure 6). The main difference among the different dyes was represented by the substituents on the phenyl ring in position 2, being both electron-withdrawing (-NO_2_ and -Cl) and electron-donating (-OMe and -NMe_2_) groups. Complex **12c** was characterized by an unsubstituted phenyl ring.

Compounds **12a**–**12e** showed fully reversible redox properties. By comparing the substituted dyes with the unsubstituted **12c,** it emerged that electron-withdrawing groups caused an increase in the redox potential (+50 mV for **12a** and +60 mV for **12b**) whereas the electron-donating groups caused the opposite effect, but in a more pronounced way (−180 mV and −190 mV for **12d** and **12e**, respectively). The effect of the different substitution was also observed in the absorption spectra, since an increase in the electron-donor properties of the benzimidazole ligand was associated with a red-shift of the longest absorption maxima of the complexes, up to 40 nm. Moreover, each dye had higher molar absorption coefficients with respect to **N719**, except in the region between 500 and 550 nm, due to both the stronger electron-donating ability and the extended π-system of cyclometalating benzimidazoles compared to isothiocyanates.

The photovoltaic performances were not measured using the dyes **12a**–**12e**, but the corresponding carboxylic acids **12f**–**12h**, obtained through basic hydrolysis using tetrabutylammonium hydroxide followed by protonation with HCl. The absorption spectra of the carboxylic acids were identical to those of the esters **12a**–**12e**. The determination of the photovoltaic properties was carried out using I^−^/I_3_^−^ redox couple, under AM 1.5G conditions. The dye **12f** showed the poorest performances because the efficient non-radiative decay of the excited state of the dye prevented the electron injection in the TiO_2_ layer. On the other hand, the other dyes demonstrated similar photovoltaic properties, and the best ones were those with the electron-donating moieties, namely complexes **12i** and **12j**. Additionally, in this case, the reference dye **N719** still gave the best results. The transit time (τ_tr_, i.e., the time necessary to cross the TiO_2_ layer and reach the FTO surface) and the lifetime of electrons (τ) were determined through intensity modulated photocurrent (IMPS) and photovoltage spectroscopy (IMVS), finding that the lifetimes of electrons were 3–4.5 times longer than the injection times (**12g**: τ_tr_ = 4.0 ms, τ = 12.6 ms, **12h**: τ_tr_ = 3.1 ms, τ = 10.7 ms, **12i**: τ_tr_ = 2.5 ms, τ = 9.3 ms, **12j**: τ_tr_ = 2.0 ms, τ = 9.3 ms). From the transit time and the lifetime of electrons it was possible to determine the charge collection efficiency (h_cc_). Each dye allowed for a high h_cc_ (**12g**: 0.68, **12h**: 0.71, **12i**: 0.73, **12j**: 0.78), making them promising candidates for applications in DSSCs.

### 2.2. Ruthenium Compounds with non Cyclometalating Ligands

Although many research groups have exploited the presence of N^C cyclometalating ligands for the ruthenium sensitizers, others have focused their attention on the use of N-based ligands; such ligands can belong to the family of N^N- or N^O-type ligands (chelating the metal via at least one anionic nitrogen atom) or can be monodentate. In this paragraph the different solutions proposed in this field will be presented and discussed.

**Figure 7:**
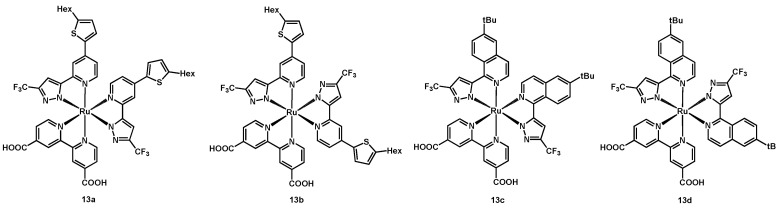
Structure of dyes **13a**–**13d**.

In 2014, Robertson, Chi et al. reported the synthesis and study of two couples of ruthenium(II) complexes [34], whose anchoring ligand was a dcbipy, while the ancillary ligands were a hexylthienyl-substituted pyrazolyl-pyridine for **13a** and **13b**, and a pyrazolyl-isoquinoline for **13c** and **13d**; moreover, the pyrazole ring carried a CF_3_ group, whereas the isoquinoline a *tert*-butyl. The structure of the complexes is shown in Figure 7.

The effect of the molecular isomerism was studied by employing the aforementioned complexes as sensitizers in DSSCs, together with two electrolyte solutions: the former consisting of 0.6 M 1,2-dimethyl-3-propylimidazolium iodide (DMPII) + 0.05 M I_2_ + 0.5 M TBP in a 15:85 (*v*/*v*) mixture of valeronitrile and acetonitrile, while the latter of a mixture of 0.6 M 1-methyl-3-propylimidazolium iodide (PMII) + 0.03 M I_2_ + 0.5 M TBP + 0.1 M guanidinium thiocyanate + 0.05 M LiI in the same solvents. The best performing dye with the first electrolyte mixture was **13c**, giving a J_sc_ of 15.23 mA cm^−2^ and a η of 9.90%, (Table 3, entry 3), followed by **13a**, with a photocurrent of 13.81 mA cm^−2^ and an efficiency of 8.87% (entry 1). The lower values provided by sensitizers **13b** and **13d** (entries 2 and 4) were attributable to the lower loading on the semiconductor and to the asymmetry of their structure, which did not allow for a uniform coverage of the TiO_2_ surface, causing an easier access to it for the I_3_^−^ species.

Considering the second electrolyte, the best performances were those achieved with **13a**, with a photocurrent and an efficiency of 18.23 mA cm^−2^ and 9.11%, respectively (entry 5). Data are summarized in Table 3.

**Figure 8:**
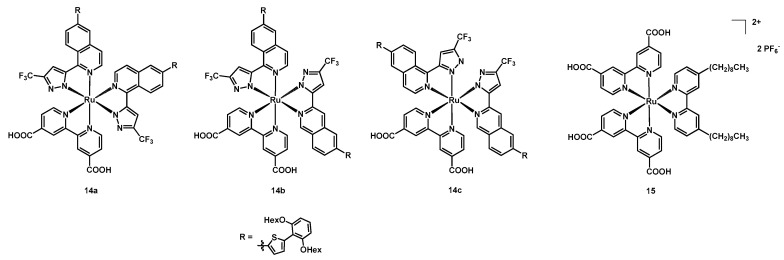
Structure of dyes **14a**–**14c** and **15**.

Additionally, in 2014, the same group published a paper on three new isomeric Ru(II) complexes [35] having the same three ligands, namely a simple anchoring dcbipy and two pyrazolyl-isoquinolines (structure in Figure 8). Although the ligands were the same, the compounds were different because of the relative disposition of the N^N ligands: while **14a** and **14c** were symmetrical, compound **14b** was not. The isomers were identified by NMR spectroscopy, especially through ^19^F spectra to distinguish them.

The chosen ligand was an evolution of those employed in the previous work for dyes **13a**–**13d**: the thienyl moiety on the isoquinoline system was introduced to expand the π conjugation of the ligand, whereas the function of the dihexyloxy-phenyl ring was to prevent aggregation of the molecules, providing larger sterical hindrance.

The dyes were tested together with three different electrolytic solutions in acetonitrile: the first having 0.6 M DMPII + 0.05 M I_2_ + 0.5 M TBP, the second 0.45 M [Co(Phen)_3_][TFSI]_2_ (TFSI = trifluoromethanesulfonyl)imide) + 0.15 M [Co(Phen)_3_][TFSI]_3_ + 0.15 M LiTFSI + 0.8 M TBP, while the third 0.45 M DMPII + 0.05 M I_2_ + 0.15 M LiI + 0.8 M TBP. Considering the first solution, compounds **14a** and **14c** gave the best results, with a J_sc_ of 12.93 and 12.41 mA cm^−2^, respectively (Table 3, entries 9 and 11), versus the 9.81 mA cm^−2^ of **14b** (entry 10). The efficiency followed also this trend, with values of 8.37%, 5.55%, and 8.26% for dyes **14a**–**c** (entries 9–11).

The same relationship was observed for the other iodine-based solution (i.e., the third one), with photocurrents of 14.49 and 14.84 vs. 10.39 mA cm^−2^ (entries 13–15). A quite different situation appeared when employing the Co^2+/3+^ redox couple (entries 16–18), since dyes **14a** and **14b** had very close values of J_sc_ (13.44 and 13.30 mA cm^−2^), V_oc_ (0.84 and 0.82 V), Fill Factor (0.757 and 0.766), and η (8.55 and 8.36%), while **14c** was the best performing, with a J_sc_ of 14.32 mA cm^−2^ and an efficiency of 9.06% (entry 18). This phenomenon was attributed to the structure of complex **14c**, having the dialkyloxy-phenyl moieties oriented towards the titania layer, thus hampering the reduction in Co(III) species with the electrons injected in the semiconductor by the sensitizer. Table 3 reports all data.

In the same year Colombo, Magni, Caramori et al. tested three copper-based redox couples with the ruthenium dye **15**, having two dcbipy anchoring ligands and a dnbipy [36]. The chosen redox mediators were [Cu(2,9-dimethyl-1,10-phenanthroline)_2_]^+/2+^ (Cu1), [Cu(2,9-dimethyl-4,7-diphenyl-1,10-phenanthroline)_2_]^+/2+^ (Cu2), and [Cu(2-mesityl-4,7-dimethyl-1,10-phenanthroline)_2_]^+/2+^ (Cu3), all obtained as hexafluorophosphate salts.

In addition, the mentioned copper(I)/(II) compounds and the typical I^−^/I_3_^−^ reference, the sensitizer was tested with an electrolytic mixture containing the iron-based co-mediators Fe1 and Fe2, where Fe1 was [Fe(dmo-bipy)_3_]^2+^ and Fe2 was [Fe(dtb-bipy)_3_]^2+^ (dmo-bipy = 4,4′-dimethoxy-2,2′-bipyridine and dtb-bipy = 4,4′-di-*tert*-butyl-2,2′-bipyridine).

**Figure 9:**
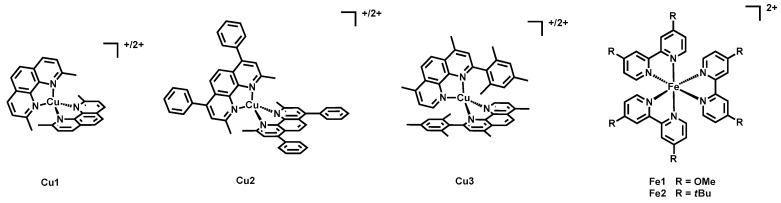
Structure of the redox mediators Cu1-Cu3 and co-mediators Fe1-Fe2.

The structure of **15** is shown in Figure 8, while that of the copper and iron shuttles is in Figure 9.

The best photocurrent (4.0 mA cm^−2^, Table 3, entry 25) was achieved by the mixture of 0.10 M Cu3 + 0.01 M Fe2, with a very similar value to that obtained using the classical iodide/triiodide redox couple (3.8 mA cm^−2^, entry 28) and an efficiency of 1.2%.

Furthermore, the highest open-circuit voltages (0.73 V, entry 19) were those provided by Cu1 (see Table 3 for all data).

**Figure 10:**
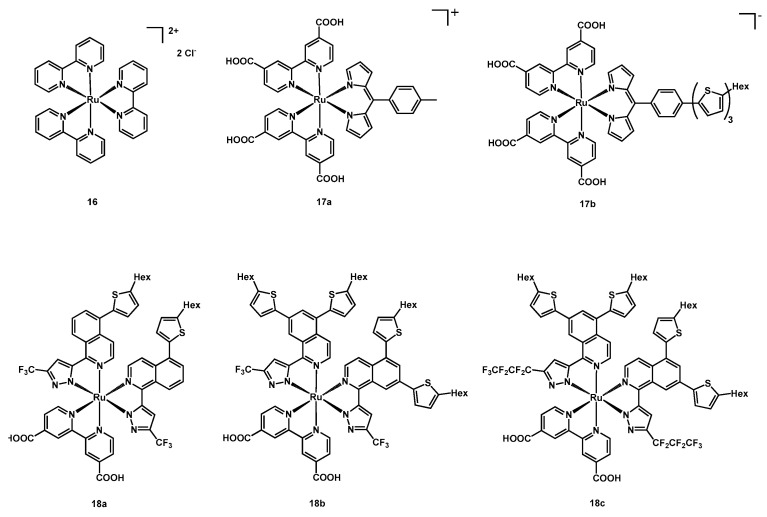
Structure of dyes **16**, **17a**–**17b,** and **18a**–**18c**.

A much simpler ruthenium(II) complex was employed by Hara et al. in 2015 [37]. In fact, the authors tested the homoleptic carboxylate-free tris(bipy)ruthenium(II) dichloride hexahydrate (**16**, Figure 10) together with the sodium salt of a carboxymethyl-𝛽-cyclodextrin (CM-𝛽-CD). This cyclodextrin had 7 glucose units and a cavity sufficiently large to host the Ru complex, thanks to hydrophobic interactions in aqueous medium; these compounds had already been tested as helpful additives in DSSC [38,39] and their use could result in better performances of the solar cells.

The formation of the host-guest structure was confirmed by luminescence measurements, since a shift and a broadening of the fluorescence spectrum were observed. Some solar cells were fabricated using **16** with and without the presence of CM-𝛽-CD; the best results were achieved by the **16**-cyclodextrine aggregate under an excitation wavelength of 490 nm, reaching an IPCE of 3.33%, a FF of 0.81 and an open-circuit voltage of 0.54 V (Table 4, entry 3). These data showed a remarkable improvement if compared with the device having only the Ru complex, due to the more difficult recombination of triiodide and electrons on the less accessible titania surface.

Two dipirrinato ruthenium(II) complexes were published in the same year by Singh and coworkers [40]. These compounds had two dcbipy ligands and a dipirrine anionic ligand, with a 4-tolyl substituent in the case of **17a** and a hexyl-bearing terthiophene in **17b** (structures in Figure 10).

These sensitizers were tested in DSSCs using an electrolyte mixture composed of DMPII + 0.05 M I_2_ + 0.1 M LiI in acetonitrile under AM 1.5G irradiation (100 mW cm^−2^).

Then, **17a** had remarkably higher performances with respect to **17b**, reaching an efficiency of 2.79% and a J_sc_ of 9.767 mA cm^−2^, to be compared to the values of 0.85% and 4.275 mA cm^−2^ of the second dye (Table 4, entries 4–5). This could be explained by the fast recombination occurring with **17b**, due to unfavorable position of the HOMO level, thus limiting the application in solar cells even if having superior panchromatic light-harvesting features.

In 2016, Chi, Nazeeruddin and coworkers published other three isocyanate-free ruthenium compounds [41], whose ancillary ligands had the basic structure of a substituted pyrazolyl-isoquinoline, as for sensitizers **13c**–**d** and **14a**–**c** previously discussed, while the anchoring ligand was still a simple dcbipy.

As can be seen in Figure 10, complex **18a** presented a CF_3_ group on the pyrazole and a hexylthiophene on the isoquinoline, while **18b** had the same ligand but with an additional Hex-thiophene on the isoquinoline. In addition, in **18c** the trifluoromethyl group was substituted with a perfluorinated *n*-butyl chain.

These dyes were tested with three different electrolyte mixtures in acetonitrile: the first was iodine-based and contained 0.45 M PMII + 0.15 M I_2_ + 0.15 M LiI + 0.8 M TBP; the second contained 0.6 M [Co(bipy)_3_][TFSI]_2_ + 0.15 M [Co(bipy)_3_][TFSI]_3_ + 0.15 M LiTFSI + 0.8 M TBP, while the third 0.6 M [Co(Phen)_3_][TFSI]_2_ + 0.15 M [Co(bipy)_3_][TFSI]_3_ + 0.15 M LiTFSI + 0.8 M TBP.

Coupled with the cobalt-phenanthroline redox mediators, **18c** gave the best results, with a J_SC_ of 13.89 mA cm^−2^, a V_OC_ of 900 mV, a FF of 0.762 and an efficiency of 9.53% (Table 4, entry 14). On the contrary, sensitizers **18a** and **18b** reached the highest photocurrents (15.31 and 14.17 mA cm^−2^, respectively, entries 6 and 7) with the I^−^/I_3_^−^ electrolytes, even if with lower efficiencies (8.20 and 7.66%). All data are listed in Table 4.

Rochford and coworkers published in 2017 an extensive study on four new 8-oxoquinolate-based Ru(II) dyes [42], whose performances were compared to those of the known sensitizers **N3** and **YE05** (structure of both compounds in Figure 1).

**Figure 11:**
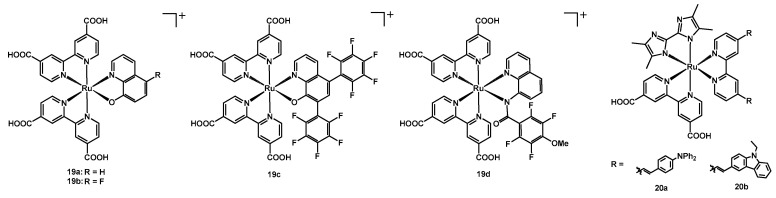
Structure of dyes **19a**–**19d** and **20a**–**20b**.

Compound **19a** had two dcbipy ligands and a simple 8-oxoquinolate, while dyes **19b**-**19d** presented more electron-withdrawing substituents on the quinoline, aiming at the reduction in the electron-donating strength of this ancillary ligand, hence improving its redox properties without limiting its light-harvesting ability. In **19b** a fluorine atom was present in position 5 of the quinoline, while in **19c** two pentafluoro-phenyl rings in positions 5 and 7. Finally, in compound **19d** the N^O ligand was replaced with a N^N one, having an amidic nitrogen donor-atom in position 8, as shown in Figure 11.

The redox properties of these complexes came from the fact that the ancillary ligands were non-innocent, since a mixing of the metal-dπ and ligand-π orbitals occurred, generating hybrid metal–ligand frontier orbitals.

The novel dyes and the two reference sensitizers were tested under 1 sun conditions (100 mW cm^−2^) using iodine-based electrolyte solutions containing 0.60 M 1,3-dimethylimidazolium iodide + 0.03 M I_2_ + 0.10 M guanidinium thiocyanate + 0.50 M TBP in a 85:15 mixture of acetonitrile and valeronitrile. Among the new dyes, **19a** and **19b** showed very poor results (Table 4, entries 15–16) when applied in solar cells, while **19d** gave the best results, with a J_sc_ of 7.25 mA cm^−2^, a V_oc_ of 0.57 V and an efficiency of 3.06% (entry 18), so approaching the values obtained by **YE05** (entry 19), but still being much less performing than **N3** (entry 20).

In the work of El-Shafei and coworkers from 2017, two new heteroleptic Ru complexes were published [43], having an anchoring dcbipy, a tetramethyl bis-imidazole and a bipyridine bearing an electron-donating moiety, namely a diphenylamino-phenyl group in the case of **20a** and a *N*-ethyl-carbazolyl moiety in **20b** (Figure 11).

Considering the short-circuit current and the efficiency, the best results in the produced solar cells were given by **20a**, with a J_sc_ of 10.20 mA cm^−2^ and a η of 3.32% (Table 4, entry 21), being around three times higher than the corresponding results of **20b** (entry 22).

**Figure 12:**
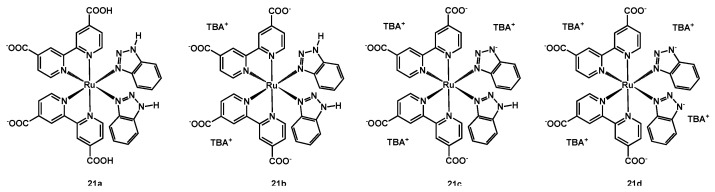
Structure of dyes **21a**–**21d**.

A different manner to reduce the recombination of the oxidized redox shuttle with the electrons on the titania surface was tested and published by Araki and coworkers in 2017 [44]; in this paper, they described a family of ruthenium(II) complexes (structure in Figure 12) having two dcbipy ligands and two benzotriazoles (btzH) ancillary ligands, whose degree of deprotonation was investigated by titration of an acidic solution of the dye by means of NaOH.

Starting from a solution of the neutral compound [Ru(dcbipyH)_2_(btzH)_2_] (**21a**, being dcbipyH the monoprotonated 2,2′-bipyridine-4,4′-dicarboxylic acid) at pH 2, the increase in pH up to a value of 4.2 gave complex **21b**, i.e., [Ru(dcbipy)_2_(btzH)_2_][TBA]_2_, then [Ru(dcbipyH)_2_(btzH)(btz)][TBA]_3_ at pH = 9 (**21c**) and finally [Ru(dcbipyH)_2_(btzH)(btz)][TBA]_4_ (**21d**) at pH = 13. All these compounds were isolated and tested in DSSCs, in order to study the effect of a growing electron density around the metal complex, coming from a higher deprotonation of both the anchoring and the ancillary ligands.

According to impedance measurements, the concept of the electrostatic blocking barrier was successfully demonstrated by the result that the highest recombination resistance was provided by complex **21d**; the trend of the resistance was the following: **21d** > **N719** > **21c** > **21b** > **21a**, including also sensitizer **N719** as reference dye.

Considering the performances of the solar cells sensitized with the mentioned Ru species, an opposite trend was observed, with **21a** giving a J_sc_ of 8.1 mA cm^−2^ and an efficiency of 2.8% (Table 4, entry 23), while **21d** a J_sc_ of 6.2 mA cm^−2^ and a η of 2.5% (entry 26).

**Figure 13:**
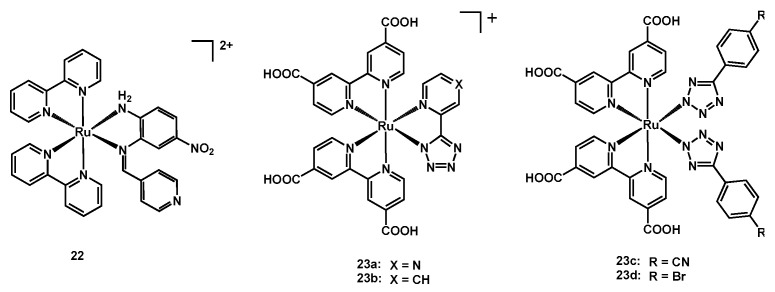
Structure of dyes **22** and **23a**–**23d**.

In 2019, a new heteroleptic complex was published by Swarnalatha and coworkers [45]. This compound (**22**, structure in Figure 13) had two bipy ligands and a neutral nitro-substituted N^N ligand binding the metal with an amine and an enamine group. The produced device had an efficiency of 3.42%, a J_sc_ of 7.12 mA cm^−2^, a V_oc_ of 0.79 V and a Fill Factor of 0.61 (Table 4, entry 28).

In 2020, Sangiorgi, Caramori, Stagni et al. published three novel ruthenium(II) sensitizers, having in common two dcbipy ligands [46]; complex **23a** had a tetrazolyl-pyrimidine (similar to the previously published complex **23b**, having a tetrazolyl-pyridine [49]), while the others had two monodentate 4-R-phenyl-tetrazole (R = CN for **23c** and Br for **23d**), bound to the metal through the deprotonated nitrogen atom (structure in Figure 13).

Table 4 lists photovoltaic data for the discussed compounds. When tested in solar cells, the best results were obtained by **23d**, with a J_sc_ of 2.55 mA cm^−2^, a FF of 0.73 and an efficiency of 1.10% (Table 4, entry 32).

**Figure 14:**
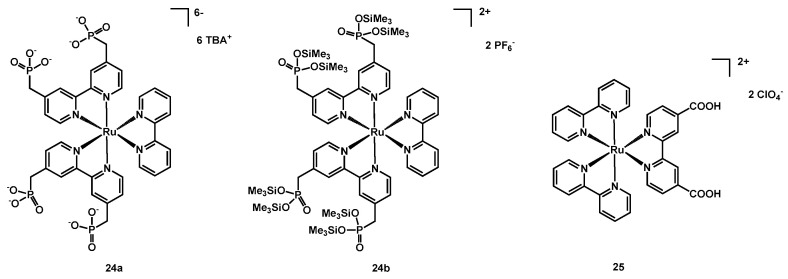
Structure of dyes **24a**–**24b** and **25**.

In the same year Jacob, Rau and coworkers presented a Ru complex (**24b**) having a simple 2,2′-bipyridine and two bipyridines substituted in positions 4 and 4′ with -CH_2_P(=O)(OSiMe_3_)_2_ chains [47]; this was a development of the similar complex **24a**, bearing -CH_2_PO_3_^2-^ chains; the structure of the two compounds is shown in Figure 14.

The dyes were tested in DSSCs having a layer of NiO over the FTO semiconductor; in fact, the phosphonate groups were the best anchoring groups on such type of metal oxide and, moreover, the methylene spacer between the pyridine ring and the phosphonate moiety improved the binding ability of the compounds.

The best performances were reached by sensitizer **24a**, with a J_sc_ of 1.6 mA cm^−2^ and an efficiency of 0.040% (Table 4, entry 34).

In 2021, Pirashanthan, Thanihaichelvan, Mariappan et al. [48] described a simple ruthenium dye (**25**, Figure 14) having two unsubstituted 2,2′-bipyridine and a 4,4′-dicarboxy-2,2′-bipyridine ligands with two perchlorate counterions. The dye was tested in DSSCs fabricated with both a conventional liquid electrolyte and with a solid one.

The absorption spectra showed a strong absorption band in the near UV region and a decay tailing around 516 nm. When compared to the reference dyes **N719**, **N3**, and **Z907**, **25** showed a higher molar absorption coefficient.

The photovoltaic performances of **25** were tested in two different manners: in the former, by employing a liquid electrolyte based on the I^−^/I_3_^−^ redox couple, in the latter having the solid electrolyte Spiro-OMeTAD. Differently from the device fabricated with the liquid electrolyte, the one containing the solid electrolyte showed a broader absorption spectrum, and the presence of the dye increased the gap between the conduction band of the TiO_2_ layer and the HOMO of the Spiro-OMeTAD from 1.0 eV to 1.4 eV, hence reducing the electron recombination. The sensitizing of TiO_2_ with **25** assisted the electron transport from Spiro-OMeTAD in the reduction in the oxidised dye to its reduced form, and acted as a hole-blocking layer. The measured J_SC_ and V_OC_ were higher in the device containing the solid electrolyte, but on the contrary the efficiency of this device was lower than that of the one fabricated with the liquid electrolyte (Table 4, entries 36–37).

## 3. Conclusions

Through this review we have shown the last developments in the research of novel thiocyanate-free bipyridine ruthenium(II) complexes to be employed as sensitizers in dye-sensitized solar cells. The compounds we have focused our attention on are characterized by a bipyridine ligand bearing or not an anchoring group such as a carboxylic moiety, through which the complex is bound to the semiconductor layer of the photoanode.

The presented bipyridine-based dyes can be divided into two categories depending on the ancillary and main light-harvesting ligand, being a N^C cyclometalating ligand in the first case and a N^N or N^O in the second one. The cyclometalated sensitizers usually share a 2-phenylpyridine or a 2-thienylpyridine, bearing different substituents or an extended aromatic system. On the other hand, the non-cyclometalated dyes often present anionic species binding the metal via nitrogen atoms, such as pyrazolyl- or tetrazolyl-pyridines, pyrazolyl-quinolines, dipyrrins, hydroxy-quinolines, bis-imidazoles, benzotriazoles.

Remarkably, efficiencies similar to that of **N719** have been reached with well designed thiocyanate-free ruthenium(II) 2,2′-bipyridyl complexes with either a cyclometalating or non-cyclometalating bidentate ancillary ligand. Particularly efficient are tris-heteroleptic cyclometalated Ru(II) complexes having a 2,2′-bipyridine-4,4′-dicarboxylic acid molecule as anchoring ligand, a bipyridine with aromatic substituents in the 4 and 4′ positions as main light-harvesting moiety and a 2,6-didodecyloxy-3,2′-bipyridine as ancillary C^N ligand, where the long alkyl chains prevent the access of the redox mediators to the semiconductor surface. Another interesting sensitizer is a Ru(II) complex bearing two 2,2′-bipyridine-4,4′-dicarboxylic acid ligands and a cyclometalating 2-phenylpyridine substituted with two CF_3_ groups on the phenyl ring, in *meta* positions with respect to the Ru-bound carbon atom, and with 5-hexylthio-3,4-ethylendioxythiophen-2-yl group in *para* position of the pyridine; its use in DSSCs led to better performances (9.03%) than those of devices produced with the classical **N719** dye (8.63%) in the same fabrication conditions. Very appealing are also ruthenium complexes bearing two phenylquinolines and a cyclometalating 2-(4-methoxyphenyl)pyridine and ruthenium complexes bearing a 2,2′-bipyridine-4,4′-dicarboxylic acid ligand and two hexylthienyl-substituted pyrazolyl-pyridines or pyrazolyl-isoquinolines, allowing efficiencies similar to that reached with **N719**.

The discussed works, which represent only a limited sample of the many families of compounds published about NCS-free ruthenium(II) dyes from the year 2014, not only demonstrate that a valid alternative to thiocyanate ligands in the aforementioned dyes is possible, but that the results can be even better than the usual reference sensitizers, paving the way to further investigations to improve the performances of the devices.

To conclude, it should be pointed out that a proper molecular design of the dye is surely essential, but it is not the only factor to be taken into account, since a suitable choice of the electrolyte mixture, of the additives and of the fabrication method is crucial to optimize the photovoltaic results and to reach in the future a wide application of such solar cells in everyday life.

## Figures and Tables

**Table 1:** Photovoltaic data of solar cells produced with Ru(II) dyes **1a**–**1b**, **2a**–**2c**, **3a**–**3d**, **4a**–**4e,** and **5a**–**5c** ^1^.

Entry	Dye ^2^	Redox Couple	V_oc_/V	J_sc_/(mA cm^−2^)	FF	η (η_rel_)/%	CE ^3^	Ref.
1	**1a** ^4^	I^−^/I_3_^− 5^	0.63	8.15	0.71	3.64 (38.8)	Pt	[16]
2	**1b** ^4^	I^−^/I_3_^− 5^	0.63	9.45	0.71	4.22 (45.0)	Pt	[16]
3	**N719** ^4^	I^−^/I_3_^− 5^	0.78	17.03	0.7	9.37	Pt	[16]
4	**2a** ^6^	Co^2+^/Co^3+ 7^	0.44	3.26	0.68	0.98	Pt	[17]
5	**2b** ^6^	Co^2+^/Co^3+ 7^	0.39	1.96	0.58	0.45	Pt	[17]
6	**2c** ^6^	Co^2+^/Co^3+ 7^	0.38	1.75	0.55	0.38	Pt	[17]
7	**Z907** ^6^	Co^2+^/Co^3+ 7^	0.4	4.86	0.72	1.33	Pt	[17]
8	**2a** ^6,8^	Co^2+^/Co^3+ 7^	0.57	7.44	0.68	2.86	Pt	[17]
9	**2b** ^6,8^	Co^2+^/Co^3+ 7^	0.51	5.53	0.69	1.94	Pt	[17]
10	**2c** ^6,8^	Co^2+^/Co^3+ 7^	0.51	5.33	0.71	1.91	Pt	[17]
11	**Z907** ^6,8^	Co^2+^/Co^3+ 7^	0.59	9.73	0.65	3.76	Pt	[17]
12	**3a** ^9^	I^−^/I_3_^− 10^	0.63	20.454	0.686	8.83	Pt	[18]
13	**3a** ^9^	I^−^/I_3_^− 11^	0.653	19.14	0.698	8.72 (94.6)	Pt	[18]
14	**3b** ^9^	I^−^/I_3_^− 10^	0.66	20.585	0.715	9.71	Pt	[18]
15	**3b** ^9^	I^−^/I_3_^− 11^	0.676	19.872	0.716	9.62 (104.3)	Pt	[18]
16	**3c** ^9^	I^−^/I_3_^− 12^	0.492	16.999	0.642	5.37	Pt	[18]
17	**3c** ^9^	I^−^/I_3_^− 10^	0.584	12.832	0.647	4.85	Pt	[18]
18	**3d** ^9^	I^−^/I_3_^− 12^	0.497	14.68	0.614	4.48	Pt	[18]
19	**3d** ^9^	I^−^/I_3_^− 10^	0.575	10.787	0.682	4.23	Pt	[18]
20	**N719** ^9^	I^−^/I_3_^− 11^	0.733	17.16	0.725	9.12	Pt	[18]
21	**N719** ^9^	I^−^/I_3_^− 11^	0.749	16.85	0.739	9.32	Pt	[18]
22	**4a** ^13^	I^−^/I_3_^− 12^	0.62	8.06	0.68	3.39	Pt	[19]
23	**4b** ^13^	I^−^/I_3_^− 12^	0.58	4.41	0.69	1.77	Pt	[19]
24	**4c** ^13^	I^−^/I_3_^− 12^	0.57	3.54	0.69	1.4	Pt	[19]
25	**4d** ^13^	I^−^/I_3_^− 12^	0.56	3.88	0.69	1.5	Pt	[19]
26	**4e** ^13^	I^−^/I_3_^− 12^	0.55	4.67	0.66	1.69	Pt	[19]
27	**5a** ^14^	I^−^/I_3_^− 15^	0.4	7.41	0.67	2.00	Pt	[20]
28	**5b** ^14^	I^−^/I_3_^− 15^	0.468	15.24	0.65	4.64	Pt	[20]
29	**5c** ^14^	I^−^/I_3_^− 15^	0.408	8.02	0.67	2.18	Pt	[20]
30	**N719** ^16^	I^−^/I_3_^− 15^	0.619	18.5	0.64	7.41	Pt	[20]
31	**5a** ^14^	I^−^/I_3_^− 17^	0.39	16.46	0.59	3.81	Pt	[20]
32	**5b** ^14^	I^−^/I_3_^− 17^	0.468	21.16	0.56	5.53	Pt	[20]
33	**5c** ^14^	I^−^/I_3_^− 17^	0.346	18.18	0.55	3.43	Pt	[20]
34	**N719** ^16^	I^−^/I_3_^− 17^	0.502	18.23	0.61	5.56	Pt	[20]

^1^ AM 1.5 simulated light source; input intensity of 100 mW cm^−2^ if not differently indicated. ^2^ having TiO_2_ as semiconductor if not differently indicated. ^3^ CE: counterelectrode.^4^ 0.3 mM dye in CH_3_CN:tBuOH 1:1. ^5^ 0.5 M LiI + 0.05 M I_2_ + 0.5 M DMPII + 0.5 M TBP in CH_3_CN (DMPII = 1,2-dimethyl-3-propylimidazolium iodide). ^6^ 0.3 mM dye + 0.3 mM CDCA in EtOH (CDCA = chenodeoxycholic acid). ^7^ 0.2 M [Co(dmbpy)_3_][TFSI]_2_ + 0.02 M [Co(dmbpy)_3_][TFSI]_3_ + 0.1 M LiTFSI + 10.0 mM CDCA in CH_3_CN (TFSI = bis(trifluoromethane)sulfonylimide). ^8^ having an ultra-thin layer of Al_2_O_3_ on the TiO_2_ semiconductor. ^9^ 0.02 M dye in 1:1:1 CH_3_CN:tBuOH:DMSO. ^10^ 0.6 M DMPII + 0.1 M LiI + 0.05 M I_2_ + 0.3 M TBP in CH_3_CN. ^11^ 0.6 M DMPII + 0.1 M LiI + 0.05 M I_2_ + 0.5 M TBP in CH_3_CN. ^12^ 0.6 M DMPII + 0.1 M LiI + 0.05 M I_2_ in CH_3_CN. ^13^ 0.2 mM dye + CDCA in 1:1 CH_3_CN:*t*BuOH. ^14^ 0.1 mM dye + 10.0 mM DCA in EtOH (DCA = deoxycholic acid). ^15^ 0.6 M DMPII + 0.1 M LiI + 0.05 M I_2_ in CH_3_CN. ^16^ 0.3 mM dye in EtOH. ^17^ 2.0 M LiI + 0.05 M I_2_ in CH_3_CN.

**Table 2:** Photovoltaic data of solar cells produced with Ru(II) dyes **6a**, **7a**–**7f**, **7a**–**7f**, **8a**–**8c**, **9c**–**9d**, **10a**–**10f**, **11a**–**11d**, **12f**–**12j** ^1^.

Entry	Dye ^2^	Redox Couple	V_oc_/V	J_sc_/(mA cm^−2^)	FF	η (η_rel_)/%	CE ^3^	Ref.
1	**P1** ^4,5^	I^−^/I_3_^− 6^	0.091	1.54	0.35	0.049	Pt	[26]
2	**P1** ^4,5^	I^−^/I_3_^− 6^	0.095	1.26	0.35	0.042	Pt	[26]
3	**P1** ^4,7^	I^−^/I_3_^− 6^	0.088	1.84	0.35	0.057	Pt	[26]
4	**P1** ^4,7^	I^−^/I_3_^− 6^	0.082	1.96	0.32	0.051	Pt	[26]
5	**6a** ^5,8^	I^−^/I_3_^− 6^	0.093	2.18	0.39	0.079	Pt	[26]
6	**6a** ^5,8^	I^−^/I_3_^− 6^	0.094	2.00	0.41	0.077	Pt	[26]
7	**6a** ^7,8^	I^−^/I_3_^− 6^	0.095	3.38	0.36	0.116	Pt	[26]
8	**6a** ^7,8^	I^−^/I_3_^− 6^	0.095	3.34	0.34	0.109	Pt	[26]
9	**7a** ^9^	Co^2+^/Co^3+ 10^	0.827	12.25	0.755	7.9	Pt-FTO	[27]
10	**7b** ^9^	Co^2+^/Co^3+ 10^	0.81	10.68	0.779	6.9	Pt-FTO	[27]
11	**7c** ^9^	Co^2+^/Co^3+ 10^	0.845	14.55	0.747	9.4	Pt-FTO	[27]
12	**7d** ^9^	Co^2+^/Co^3+ 10^	0.794	9.89	0.785	6.3	Pt-FTO	[27]
13	**7e** ^9^	Co^2+^/Co^3+ 10^	0.794	11.28	0.769	7.0	Pt-FTO	[27]
14	**7f** ^9^	Co^2+^/Co^3+ 11^	0.807	11.85	0.736	7.2	Pt-FTO	[27]
15	**7a** ^9^	I^−^/I_3_^− 12^	0.642	16.16	0.624	6.6	Pt-FTO	[27]
16	**7b** ^9^	I^−^/I_3_^− 13^	0.57	13.98	0.718	5.8	Pt-FTO	[27]
17	**7c** ^9^	I^−^/I_3_^− 13^	0.647	15.32	0.706	7.1	Pt-FTO	[27]
18	**7d** ^9^	I^−^/I_3_^− 13^	0.694	13.84	0.738	7.2	Pt-FTO	[27]
19	**7e** ^9^	I^−^/I_3_^− 12^	0.649	15.77	0.668	7.0	Pt-FTO	[27]
20	**7f** ^9^	I^−^/I_3_^− 13^	0.634	14.71	0.699	6.7	Pt-FTO	[27]
21	**7b** ^9^	I^−^/I_3_^− 13^	0.57	13.98	0.624	6.6	Pt-FTO	[28]
22	**7c** ^9^	I^−^/I_3_^− 13^	0.647	15.32	0.706	7.1	Pt-FTO	[28]
23	**7d** ^9^	I^−^/I_3_^− 13^	0.694	13.84	0.738	7.2	Pt-FTO	[28]
24	**7f** ^9^	I^−^/I_3_^− 13^	0.634	14.71	0.699	6.7	Pt-FTO	[28]
25	**8a** ^14,15^	Co^2+^/Co^3+ 16^	0.819	13.68	0.715	8.0	Pt- FTO	[29]
26	**8b** ^14,15^	Co^2+^/Co^3+ 16^	0.845	13.89	0.7	8.2	Pt- FTO	[29]
27	**8c** ^14,15^	Co^2+^/Co^3+ 16^	0.809	13.03	0.721	7.6	Pt- FTO	[29]
28	**9c** ^17^	- ^18^	0.65	18	0.46	5.3	PECC-2 ^19^	[30]
29	**9d** ^17^	- ^18^	0.58	10.7	0.65	4.1	PECC-2 ^19^	[30]
30	**N3** ^17^	- ^18^	0.71	16.5	0.53	6.1	PECC-2 ^19^	[30]
31	**10a** ^20^	I^−^/I_3_^− 21^	0.733	15.01	0.67	7.40 (85.7)	Pt-FTO	[31]
32	**10b** ^20^	I^−^/I_3_^− 21^	0.737	18.4	0.67	9.03 (104.6)	Pt-FTO	[31]
33	**10c** ^20^	I^−^/I_3_^− 21^	0.737	14.29	0.67	7.01 (81.2)	Pt-FTO	[31]
34	**10d** ^20^	I^−^/I_3_^− 21^	0.741	17.74	0.68	8.92 (103.4)	Pt-FTO	[31]
35	**10e** ^20^	I^−^/I_3_^− 21^	0.391	0.13	0.42	0.02 (0.23)	Pt-FTO	[31]
36	**10f** ^20^	I^−^/I_3_^− 21^	0.381	0.13	0.42	0.02 (0.23)	Pt-FTO	[31]
37	**N719** ^20^	I^−^/I_3_^− 21^	0.744	17.54	0.66	8.63	Pt-FTO	[31]
38	**11a** ^20^	I^−^/I_3_^− 21^	0.57	14.9	0.71	6.00 (82.6)	Pt-FTO	[32]
39	**11b** ^20^	I^−^/I_3_^− 21^	0.6	15.4	0.71	6.54 (90.1)	Pt-FTO	[32]
40	**11c** ^20^	I^−^/I_3_^− 21^	0.6	15.6	0.69	6.49 (89.4)	Pt-FTO	[32]
41	**11d** ^20^	I^−^/I_3_^− 21^	0.61	16.5	0.69	6.97 (96.0)	Pt-FTO	[32]
42	**N719** ^20^	I^−^/I_3_^− 21^	0.67	14.8	0.73	7.26	Pt-FTO	[32]
43	**12f** ^17^	I^−^/I_3_^− 22^	0.28	0.32	0.58	0.05 (1.02)	PECC-2 ^19^	[33]
44	**12g** ^17^	I^−^/I_3_^− 22^	0.61	2.8	0.84	1.45 (29.5)	PECC-2 ^19^	[33]
45	**12h** ^17^	I^−^/I_3_^− 22^	0.62	3.3	0.81	1.65 (33.5)	PECC-2 ^19^	[33]
46	**12i** ^17^	I^−^/I_3_^− 22^	0.61	4.6	0.8	2.25 (45.7)	PECC-2 ^19^	[33]
47	**12j** ^17^	I^−^/I_3_^− 22^	0.61	6	0.8	2.93 (59.5)	PECC-2 ^19^	[33]
48	**N719** ^17^	I^−^/I_3_^− 22^	0.75	8.2	0.8	4.92	PECC-2 ^19^	[33]

^1^ AM 1.5 simulated light source; input intensity of 100 mW cm^−2^ if non differently indicated. ^2^ having TiO_2_ as semiconductor if not differently indicated. ^3^ CE: counterelectrode. ^4^ 0.3 mM dye in CH_3_CN. ^5^ having 1 layer of NiO as semiconductor. ^6^ 1.0 M LiI + 0.1 M I_2_ in CH_3_CN. ^7^ having 2 layers of NiO as semiconductor. ^8^ 0.1 mM dye in EtOH. ^9^ 0.2 mM dye in 3:7 THF:EtOH. ^10^ 0.25 M [Co(Phen)_3_][TFSI]_2_ + 0.05 M [Co(Phen)_3_][TFSI]_3_ + 0.1 M LiTFSI + 0.25 M NP (NP = 4-(5-nonyl)pyridine). ^11^ 0.25 M [Co(Phen)_3_][TFSI]_2_ + 0.05 M [Co(Phen)_3_][TFSI]_3_ + 0.1 M LiTFSI + 0.50 M NP. ^12^ 1.0 M PMII + 0.10 M LiI + 0.03 M I_2_ + 0.5 M TBP + 0.1 M GNCS) in CH_3_CN (PMII = 1-methyl-3-propylimidazolium iodide, GNCS = Guanidinium thiocyanate). ^13^ 1.0 M PMII + 0.05 M LiI + 0.03 M I_2_ + 0.5 M TBP + 0.1 M GNCS in CH_3_CN. ^14^ 4.5 μm + 5 μm double layer of TiO_2_. ^15^ 0.2 mM dye in THF:EtOH 1:4. ^16^ 0.25 M [Co(Phen)_3_][TFSI]_2_ + 0.05 M [Co(Phen)_3_][TFSI]_3_ + 0.25 M TBP + 0.1 M LiTFSI in CH_3_CN. ^17^ 0.5 mM in MeOH. ^18^ the dye regeneration process was studied using the photopotential and photocurrent transient methods. ^19^ PECC: photoelectrochemical cell (Zahner), Pt working electrode + Pt wire with the surface area of 5 cm^2^ as an auxiliary electrode + Ag wire as reference electrode. ^20^ 0.2 mM dye + 2–30 mM CDCA in 1:1:1 DMSO:ACN:*t*BuOH. ^21^ 0.6 M BMII + 0.2 M LiI + 0.03 M I_2_ + 0.5 M TBP + 0.1 M GNCS in CH_3_CN (BMII = 1-butyl-3-methylimidazolium iodide). ^22^ 0.5 M LiI + 0.05 M I_2_ in CH_3_CN.

**Table 3:** Photovoltaic data of solar cells produced with Ru(II) dyes **13a**–**13d**, **14a**–**14c**, and **15** ^1^.

Entry	Dye ^2^	Redox Couple	V_oc_/V	J_sc_/(mA cm^−2^)	FF	η (η_rel_)/%	CE ^3^	Ref.
1	**13a** ^4^	I^−^/I_3_^− 5^	0.83	13.81	0.774	8.87	Pt	[34]
2	**13b** ^4^	I^−^/I_3_^− 5^	0.78	10.63	0.785	6.51	Pt	[34]
3	**13c** ^4^	I^−^/I_3_^− 5^	0.85	15.23	0.764	9.90	Pt	[34]
4	**13d** ^4^	I^−^/I_3_^− 5^	0.78	11.42	0.789	7.02	Pt	[34]
5	**13a** ^4^	I^−^/I_3_^− 6^	0.72	18.23	0.694	9.11	Pt	[34]
6	**13b** ^4^	I^−^/I_3_^− 6^	0.68	14.98	0.724	7.38	Pt	[34]
7	**13c** ^4^	I^−^/I_3_^− 6^	0.72	17.63	0.702	8.92	Pt	[34]
8	**13d** ^4^	I^−^/I_3_^− 6^	0.69	14.80	0.729	7.45	Pt	[34]
9	**14a** ^7,8^	I^−^/I_3_^− 9^	0.89	12.93	0.727	8.37	Pt	[35]
10	**14b** ^7,8^	I^−^/I_3_^− 9^	0.78	9.81	0.725	5.55	Pt	[35]
11	**14c** ^7,8^	I^−^/I_3_^− 9^	0.88	12.41	0.756	8.26	Pt	[35]
12	**14a + c** ^7,8^	I^−^/I_3_^− 9^	0.87	13.12	0.731	8.34	Pt	[35]
13	**14a** ^7,8^	I^−^/I_3_^− 10^	0.78	14.49	0.668	7.55	Pt	[35]
14	**14b** ^7,8^	I^−^/I_3_^− 10^	0.68	10.39	0.681	4.80	Pt	[35]
15	**14c** ^7,8^	I^−^/I_3_^− 10^	0.73	14.84	0.651	7.06	Pt	[35]
16	**14a** ^7,8^	Co^2+^/Co^3+ 11^	0.84	13.44	0.757	8.55	Pt	[35]
17	**14b** ^7,8^	Co^2+^/Co^3+ 11^	0.82	13.30	0.766	8.36	Pt	[35]
18	**14c** ^7,8^	Co^2+^/Co^3+ 11^	0.84	14.32	0.754	9.06	Pt	[35]
19	**15** ^12^	Cu^+^/Cu^2+ 13^	0.73	1.1	0.50	0.4	PEDOT-FTO	[36]
20	**15** ^12^	Cu^+^/Cu^2+ 14^	0.72	0.9	0.53	0.4	PEDOT-FTO	[36]
21	**15** ^12^	Cu^+^/Cu^2+ 15^	0.71	1.1	0.49	0.4	PEDOT-FTO	[36]
22	**15** ^12^	Cu^+^/Cu^2+ 16^	0.58	2.4	0.58	0.9	PEDOT-FTO	[36]
23	**15** ^12^	Cu^+^/Cu^2+ 17^	0.60	2.0	0.64	0.9	PEDOT-FTO	[36]
24	**15** ^12^	Cu^+^/Cu^2+ 18^	0.55	2.7	0.49	0.8	PEDOT-FTO	[36]
25	**15** ^12^	Cu^+^/Cu^2+ 19^	0.51	4.0	0.51	1.2	PEDOT-FTO	[36]
26	**15** ^12^	Cu^+^/Cu^2+ 20^	0.62	1.7	0.69	0.8	PEDOT-FTO	[36]
27	**15** ^12^	Cu^+^/Cu^2+ 21^	0.67	1.3	0.50	0.5	PEDOT-FTO	[36]
28	**15** ^12^	I^−^/I_3_^− 22^	0.60	3.8	0.66	1.7	PEDOT-FTO	[36]
29	**15** ^12^	Cu^+^/Cu^2+ 23^	0.55	2.4	0.66	1.0	PEDOT-FTO	[36]
30	**15** ^12^	Cu^+^/Cu^2+ 24^	0.56	2.4	0.64	1.0	PEDOT-FTO	[36]
31	**15** ^12^	Cu^+^/Cu^2+ 25^	0.57	2.4	0.61	0.9	PEDOT-FTO	[36]
32	**15** ^12^	Cu^+^/Cu^2+ 26^	0.59	2.2	0.66	0.9	PEDOT-FTO	[36]
33	**15** ^12^	Cu^+^/Cu^2+ 27^	0.57	1.8	0.65	0.7	PEDOT-FTO	[36]
34	**15** ^12^	Cu^+^/Cu^2+ 28^	0.59	2.4	0.64	1.0	PEDOT-FTO	[36]
35	**15** ^12^	Cu^+^/Cu^2+ 29^	0.61	2.1	0.65	0.9	PEDOT-FTO	[36]
36	**15** ^12^	Cu^+^/Cu^2+ 30^	0.61	2.1	0.66	0.9	PEDOT-FTO	[36]
37	**15** ^12^	Cu^+^/Cu^2+ 31^	0.62	2.2	0.65	1.0	PEDOT-FTO	[36]

^1^ AM 1.5 simulated light source; input intensity of 100 mW cm^−2^ for dyes **9a**–**9d** and **10a**–**10c**; input intensity of 90 mW cm^−2^ for dye **11**. ^2^ having TiO_2_ as semiconductor if not differently indicated. ^3^ CE: counterelectrode; PEDOT = poly(3,4-ethylendioxythiophene). ^4^ 0.3 mM dye + 0.6 mM TBADC in EtOH:DMSO 4:1 (TBADC = Tetrabutylammonium deoxycholate). ^5^ 0.6 M DMPII + 0.05 M I_2_ + 0.5 M TBP in 85:15 CH_3_CN:BuCN. ^6^ 0.6 M PMII + 0.1 M LiI + 0.03 M I_2_ + 0.5 M TBP + 0.1 M GNCS in 85:15 CH_3_CN:BuCN. ^7^ having two layers of TiO_2_ as semiconductor. ^8^ 0.3 mM dye + 0.6 mM TBADC in EtOH:DMSO 9:1. ^9^ 0.6 M DMPII + 0.05 M I_2_ + 0.5 M TBP in CH_3_CN. ^10^ 0.45 M DMPII + 0.15 M LiI + 0.05 M I_2_ + 0.8 M TBP in CH_3_CN. ^11^ 0.45 M [Co(Phen)_3_][TFSI]_2_ + 0.15 M [Co(Phen)_3_][TFSI]_3_ + 0.15 M LiTFSI + 0.8 M TBP in CH_3_CN. ^12^ 0.1 mM dye, then 0.1% APTES in toluene (APTES = (3-aminopropyl)triethoxysilane). ^13^ 0.15 M Cu1 + NOBF_4_ + 0.1 M LiClO_4_ in CH_3_CN, Cu^2+^/(Cu^2+^ + Cu^+^) = 0.05. ^14^ 0.10 M Cu1 + NOBF_4_ + 0.1 M LiClO_4_ in CH_3_CN, Cu^2+^/(Cu^2+^ + Cu^+^) = 0.05. ^15^ 0.15 M Cu2 + NOBF_4_ + 0.1 M LiClO_4_ in CH_3_CN, Cu^2+^/(Cu^2+^ + Cu^+^) = 0.05. ^16^ 0.15 M Cu3 + NOBF_4_ + 0.1 M LiClO_4_ in CH_3_CN, Cu^2+^/(Cu^2+^ + Cu^+^) = 0.05. ^17^ 0.10 M Cu3 + NOBF_4_ + 0.1 M LiClO_4_ in CH_3_CN, Cu^2+^/(Cu^2+^ + Cu^+^) = 0.05. ^18^ 0.10 M Cu3 + NOBF_4_ + 0.1 M LiClO_4_ + 0.01 M Fe1 in CH_3_CN Cu^2+^/(Cu^2+^ + Cu^+^) = 0.05. ^19^ 0.10 M Cu3 + NOBF_4_ + 0.1 M LiClO_4_ + 0.01 M Fe2 in CH_3_CN, Cu^2+^/(Cu^2+^ + Cu^+^) = 0.05. ^20^ 0.10 M Cu3 + NOBF_4_ + 0.1 M LiClO_4_ + 0.1 M TBP + 0.01 M Fe2 in CH_3_CN, Cu^2+^/(Cu^2+^ + Cu^+^) = 0.05. ^21^ 0.10 M Cu1 + NOBF_4_ + 0.1 M LiClO_4_ + 0.01 M Fe2 in CH_3_CN, Cu^2+^/(Cu^2+^ + Cu^+^) = 0.05. ^22^ 0.1 M LiI in CH_3_CN. ^23^ 0.20 Cu3 + NOBF_4_ + 0.1 M LiClO_4_ in CH_3_CN, Cu^2+^/(Cu^2+^ + Cu^+^) = 0.15. ^24^ 0.20 Cu3 + NOBF_4_ + 0.1 M LiClO_4_ in CH_3_CN, Cu^2+^/(Cu^2+^ + Cu^+^) = 0.10. ^25^ 0.20 Cu3 + NOBF_4_ + 0.1 M LiClO_4_ in CH_3_CN, Cu^2+^/(Cu^2+^ + Cu^+^) = 0.05. ^26^ 0.15 Cu3 + NOBF_4_ + 0.1 M LiClO_4_ in CH_3_CN, Cu^2+^/(Cu^2+^ + Cu^+^) = 0.15. ^27^ 0.15 Cu3 + NOBF_4_ + 0.1 M LiClO_4_ in CH_3_CN, Cu^2+^/(Cu^2+^ + Cu^+^) = 0.10. ^28^ 0.15 Cu3 + NOBF_4_ + 0.1 M LiClO_4_ in CH_3_CN, Cu^2+^/(Cu^2+^ + Cu^+^) = 0.05. ^29^ 0.10 Cu3 + NOBF_4_ + 0.1 M LiClO_4_ in CH_3_CN, Cu^2+^/(Cu^2+^ + Cu^+^) = 0.15. ^30^ 0.10 Cu3 + NOBF_4_ + 0.1 M LiClO_4_ in CH_3_CN, Cu^2+^/(Cu^2+^ + Cu^+^) = 0.10. ^31^ 0.10 Cu3 + NOBF_4_ + 0.1 M LiClO_4_ in CH_3_CN, Cu^2+^/(Cu^2+^ + Cu^+^) = 0.05.

**Table 4:** Photovoltaic data of solar cells produced with Ru(II) dyes **16**, **17a**–**17b**, **18a**–**18c**, **19a**–**19d**, **20a**–**20b**, **21a**–**21b**, **22**, **23a**–**23d**, **24a**–**24b**, and **25** ^1^.

Entry	Dye ^2^	Redox Couple	V_oc_/V	J_sc_/(mA cm^−2^)	FF	η (η_rel_)/%	CE ^3^	Ref.
1	**16** ^4,5,6^	I^−^/I_3_^− 7^	0.28	0.012	0.49	0.77	Pt-FTO	[37]
2	**16** ^4,5,6,8^	I^−^/I_3_^− 7^	0.52	0.035	0.78	2.35	Pt-FTO	[37]
3	**16** ^4,5,8,9^	I^−^/I_3_^− 7^	0.54	0.062	0.81	3.33	Pt-FTO	[37]
4	**17a** ^10^	I^−^/I_3_^− 11^	0.435	9.767	0.651	2.79	Pt-FTO	[40]
5	**17b** ^10^	I^−^/I_3_^− 11^	0.320	4.275	0.619	0.85	Pt-FTO	[40]
6	**18a** ^12^	I^−^/I_3_^− 13^	0.718	15.31	0.746	8.20	FTO	[41]
7	**18b** ^12^	I^−^/I_3_^− 13^	0.727	14.17	0.743	7.66	FTO	[41]
8	**18c** ^12^	I^−^/I_3_^− 13^	0.740	13.53	0.749	7.50	FTO	[41]
9	**18a** ^12^	Co^2+^/Co^3+ 14^	0.840	12.78	0.764	8.22	FTO	[41]
10	**18b** ^12^	Co^2+^/Co^3+ 14^	0.844	13.56	0.742	8.49	FTO	[41]
11	**18c** ^12^	Co^2+^/Co^3+ 14^	0.853	13.36	0.750	8.55	FTO	[41]
12	**18a** ^12^	Co^2+^/Co^3+ 15^	0.842	12.17	0.750	7.69	FTO	[41]
13	**18b** ^12^	Co^2+^/Co^3+ 15^	0.898	12.32	0.754	8.34	FTO	[41]
14	**18c** ^12^	Co^2+^/Co^3+ 15^	0.900	13.89	0.762	9.53	FTO	[41]
15	**19a** ^16^	I^−^/I_3_^− 17^	0.45	1.18	0.64	0.34	Pt-FTO	[42]
16	**19b** ^16^	I^−^/I_3_^− 17^	0.43	1.35	0.60	0.35	Pt-FTO	[42]
17	**19c** ^16^	I^−^/I_3_^− 17^	0.56	5.93	0.69	2.23	Pt-FTO	[42]
18	**19d** ^16^	I^−^/I_3_^− 17^	0.57	7.25	0.74	3.06	Pt-FTO	[42]
19	**YE05** ^16^	I^−^/I_3_^− 17^	0.56	9.42	0.69	3.64	Pt-FTO	[42]
20	**N3** ^16^	I^−^/I_3_^− 17^	0.62	15.40	0.60	5.72	Pt-FTO	[42]
21	**20a** ^18^	I^−^/I_3_^− 19^	0.58	10.20	0.56	3.32	Pt	[43]
22	**20b** ^18^	I^−^/I_3_^− 19^	0.52	3.52	0.58	1.06	Pt	[43]
23	**21a** ^20^	I^−^/I_3_^− 21^	0.56	8.1	0.610	2.8 (49.1)	Pt-FTO	[44]
24	**21b** ^20^	I^−^/I_3_^− 21^	0.55	7.1	0.624	2.5 (43.8)	Pt-FTO	[44]
25	**21c** ^20^	I^−^/I_3_^− 21^	0.60	6.5	0.665	2.6 (45.6)	Pt-FTO	[44]
26	**21d** ^20^	I^−^/I_3_^− 21^	0.61	6.2	0.670	2.5 (43.8)	Pt-FTO	[44]
27	**N719** ^20^	I^−^/I_3_^− 21^	0.67	13.3	0.642	5.7	Pt-FTO	[44]
28	**22** ^22^	I^−^/I_3_^− 23^	0.79	7.12	0.61	3.42	Pt-FTO	[45]
29	**23a** ^24^	I^−^/I_3_^− 25^	0.548	1.30	0.72	0.50 (8.62)	FTO ^26^	[46]
30	**23b** ^24^	I^−^/I_3_^− 25^	0.564	2.15	0.70	0.87 (15.00)	FTO ^26^	[46]
31	**23c** ^24^	I^−^/I_3_^− 25^	0.546	1.35	0.68	0.52 (8.96)	FTO ^26^	[46]
32	**23d** ^24^	I^−^/I_3_^− 25^	0.592	2.55	0.73	1.10 (18.96)	FTO ^26^	[46]
33	**N719** ^24^	I^−^/I_3_^− 25^	0.693	11.70	0.71	5.80	FTO ^26^	[46]
34	**24a** ^27,28,29^	I^−^/I_3_^− 30^	0.064	1.6	0.31	0.040	Pt	[47]
35	**24b** ^27,28,31^	I^−^/I_3_^− 30^	0.081	1.1	0.23	0.026	Pt	[47]
36	**25** ^32^	I^−^/I_3_^− 33^	0.6	5.82	0.52	1.82	Pt	[48]
37	**25** ^32^	Spiro-OMeTAD ^33^	0.68	3.04	0.6	1.26	Au	[48]

^1^ AM 1.5 simulated light source; input intensity of 100 mW cm^−2^. ^2^ having TiO_2_ as semiconductor if not differently indicated. ^3^ CE: counterelectrode. ^4^ 0.03 M dye in EtOH. ^5^ Power intensity non specified. ^6^ Excitation wavelength of 450 nm. ^7^ Electrolyte formulation non specified. ^8^ With cyclodextrin solution (0.029 g/mL in water). ^9^ Excitation wavelength of 490 nm. ^10^ 0.3 mM dye + 0.02 M DCA in 1:1 CH_3_CN:*n*BuOH. ^11^ DMPII (unspecified concentration) + 0.1 M LiI + 0.05 M I_2_ in CH_3_CN. ^12^ 0.3 mM dye in 4:1 EtOH:DMSO. ^13^ 0.45 M PMII + 0.15 M LiI + 0.15 M I_2_ + 0.8 M TBP in CH_3_CN. ^14^ 0.6 M [Co(bipy)_3_][TFSI]_2_ + 0.15 M [Co(bipy)_3_][TFSI]_3_ + 0.15 M LiTFSI + 0.8 M TBP in CH_3_CN. ^15^ 0.6 M [Co(Phen)_3_][TFSI]_2_ + 0.15 M [Co(Phen)_3_][TFSI]_3_ + 0.15 M LiTFSI + 0.8 M TBP in CH_3_CN. ^16^ 0.25 mM dye in MeOH. ^17^ 0.6 M DBII + 0.05 M LiI + 0.03 M I_2_ in 85:15 CH_3_CN:*n*BuCN (DBII = 1,3-dibutylimidazolium iodide). ^18^ 0.3 mM dye + 0.04 M DCA in 1:1:1 CH_3_CN:*t*BuOH:DMSO. ^19^ Solaronix Iodolyte AN-50. ^20^ 0.12 mM dye in 3:1 CH_3_CN:*t*BuOH. ^21^ 0.6 M TBAI + 0.1 M LiI + 0.1 M I_2_ + 0.5 M TBP in 3-methoxypropionitrile (TBAI = tetrabutylammonium iodide). ^22^ 0.3 mM dye in MeOH. ^23^ 0.6 M DMPII + 0.1 M LiI + 0.05 M I_2_ + 1.0 M TBP in CH_3_CN. ^24^ 0.3 mM dye in EtOH. ^25^ 0.6 M PMII + 0.1 M LiI + 0.05 M MgI_2_ + 0.1 M I_2_ in CH_3_CN. ^26^ Not specified if with Pt or PEDOT. ^27^ having NiO as semiconductor. ^28^ 450 nm light, power not specified. ^29^ 0.5 mM dye in MeOH. ^30^ 0.1 M TBAI + 0.1 M LiI + 0.05 M I_2_ + 0.4 M TBP in CH_3_CN. ^31^ 0.5 mM dye in CH_3_CN or DCM. ^32^ 0.3 mM dye in CH_3_CN:tBuOH 1:1. ^33^ solutions A (97 mg/mL of Spiro-OMeTAD in chlorobenzene), B (175 mg/mL of LiTFSI in CH_3_CN) and C (46.6% *v*/*v* solution of TBP in CH_3_CN) were prepared separately. 1200 μL of solution A + 36.24 μL of solution B + 11.7 μL of solution C were mixed and deposited over the substrate through spin coating; the substrate was then sintered under nitrogen.

## Data Availability

No new data were created.

## References

[1] O’Regan B., Grätzel M. (1991). A low-cost, high-efficiency solar cell based on dye-sensitized colloidal TiO2 films. Nature.

[2] Yella A., Lee H.-W., Tsao H.N., Yi C., Chandiran A.K., Nazeeruddin K., Diau E.W.-G., Yeh C.-Y., Zakeeruddin S.M., Grätzel M. (2011). Porphyrin-Sensitized Solar Cells with Cobalt (II/III)–Based Redox Electrolyte Exceed 12 Percent Efficiency. Science.

[3] Magni M., Biagini P., Colombo A., Dragonetti C., Roberto D., Valore A. (2016). Versatile copper complexes as a convenient springboard for both dyes and redox mediators in dye sensitized solar cells. Coord. Chem. Rev..

[4] Freitag M., Giordano F., Yang W., Pazoki M., Hao Y., Zietz B., Grätzel M., Hagfeldt A., Boschloo G. (2016). Copper Phenanthroline as a Fast and High-Performance Redox Mediator for Dye-Sensitized Solar Cells. J. Phys. Chem. C.

[5] Magni M., Giannuzzi R., Colombo A., Cipolla M.P., Dragonetti C., Caramori S., Carli S., Grisorio R., Suranna G.P., Bignozzi C.A. (2016). Tetracoordinated Bis-phenanthroline Copper-Complex Couple as Efficient Redox Mediators for Dye Solar Cells. Inorg. Chem..

[6] Benazzi E., Magni M., Colombo A., Dragonetti C., Caramori S., Bignozzi C.A., Grisorio R., Suranna G.P., Cipolla M.P., Manca M. (2018). Bis(1,10-phenanthroline) copper complexes with tailored molecular architecture: From electrochemical features to application as redox mediators in dye-sensitized solar cells. Electrochim. Acta.

[7] Chen K.Y., Schauer P.A., Patrick B.O., Berlinguette C.P. (2018). Correlating cobalt redox couples to photovoltage in the dye-sensitized solar cell. Dalton Trans..

[8] Saygili Y., Stojanovic M., Flores-Díaz N., Zakeeruddin S.M., Vlachopoulos N., Grätzel M., Hagfeldt A. (2019). Metal Coordination Complexes as Redox Mediators in Regenerative Dye-Sensitized Solar Cells. Inorganics.

[9] Higashino T., Iiyama H., Nimura S., Kurumisawa Y., Imahori H. (2020). Effect of Ligand Structures of Copper Redox Shuttles on Photovoltaic Performance of Dye-Sensitized Solar Cells. Inorg. Chem..

[10] Colombo A., Dragonetti C., Roberto D., Fagnani F. (2021). Copper Complexes as Alternative Redox Mediators in Dye-Sensitized Solar Cells. Molecules.

[11] Wu J.H., Lan Z., Lin J.M., Huang M.L., Huang Y.F., Yunfang H., Luo G.H. (2015). Electrolytes in Dye-Sensitized Solar Cells. Chem. Rev..

[12] Colombo A., Dragonetti C., Valore A., Coluccini C., Manfredi N., Abbotto A. (2014). Thiocyanate-free ruthenium(II) 2,2′-bipyridyl complexes for dye-sensitized solar cells. Polyhedron.

[13] Carella A., Borbone F., Centore R. (2018). Research Progress on Photosensitizers for DSSC. Front. Chem..

[14] Alhorani S., Kumar S., Genwa M., Meena P.L. (2020). Review of latest efficient sensitizer in dye-sensitized solar cells. AIP Conf. Proc..

[15] Castillo-Robles J.A., Rocha-Rangel E., Ramírez-De-León J.A., Caballero-Rico F.C., Armendáriz-Mireles E.N. (2021). Advances on Dye-Sensitized Solar Cells (DSSCs) Nanostructures and Natural Colorants: A Review. J. Compos. Sci..

[16] Li C.-Y., Su C., Wang H.-H., Kumaresan P., Hsu C.-H., Lee I.-T., Chang W.-C., Tingare Y.S., Li T.-Y., Lin C.-F. (2014). Design and development of cyclometalated ruthenium complexes containing thiophenyl-pyridine ligand for dye-sensitized solar cells. Dye. Pigment..

[17] Soman S., Xie Y., Hamann T.W. (2014). Cyclometalated sensitizers for DSSCs employing cobalt redox shuttles. Polyhedron.

[18] Hussain M., Islam A., Bedja I., Gupta R.K., Han L., El-Shafei A. (2014). A comparative study of Ru(ii) cyclometallated complexes versus thiocyanated heteroleptic complexes: Thermodynamic force for efficient dye regeneration in dye-sensitized solar cells and how low could it be?. Phys. Chem. Chem. Phys..

[19] Siu C.-H., Ho C.-L., He J., Chen T., Majumda P., Zhao J., Li H., Wong W.-Y. (2014). Optimizing the photovoltaic performance of thiocyanate-free ruthenium photosensitizers by structural modification of C^N cyclometalating ligand in dye-sensitized solar cells. Polyhedron.

[20] Funaki T., Kusama H., Onozawa-Komatsuzaki N., Kasuga K., Sayama K., Sugihara H. (2014). Near-IR Sensitization of Dye-Sensitized Solar Cells Using Thiocyanate-Free Cyclometalated Ruthenium(II) Complexes Having a Pyridylquinoline Ligand. Eur. J. Inorg. Chem..

[21] Bomben P.G., Robson K.C.D., Sedach P.A., Berlinguette C.P. (2009). On the Viability of Cyclometalated Ru(II) Complexes for Light-Harvesting Applications. Inorg. Chem..

[22] Bomben P.G., Koivisto B.D., Berlinguette C.P. (2010). Cyclometalated Ru Complexes of Type [RuII(N∧N)2(C∧N)]z: Physicochemical Response to Substituents Installed on the Anionic Ligand. Inorg. Chem..

[23] Robson K.C.D., Bomben P.G., Berlinguette C.P. (2012). Cycloruthenated sensitizers: Improving the dye-sensitized solar cell with classical inorganic chemistry principles. Dalton Trans..

[24] Bomben P.G., Borau-Garcia J., Berlinguette C.P. (2012). Three is not a crowd: Efficient sensitization of TiO2 by a bulky trichromic trisheteroleptic cycloruthenated dye. Chem. Commun..

[25] Pogozhev D.V., Bezdek M.J., Schauer P.A., Berlinguette C.P. (2013). Ruthenium(II) Complexes Bearing a Naphthalimide Fragment: A Modular Dye Platform for the Dye-Sensitized Solar Cell. Inorg. Chem..

[26] Brunner F., Marinakis N., Wobill C., Willgert M., Ertl C.D., Kosmalski T., Neuburger M., Bozic-Weber B., Glatzel T., Constable E.C. (2016). Modular synthesis of simple cycloruthenated complexes with state-of-the-art performance in p-type DSCs. J. Mater. Chem. C.

[27] Aghazada S., Gao P., Yella A., Marotta G., Moehl T., Teuscher J., Moser J.-E., De Angelis F., Grätzel M., Nazeeruddin M.K. (2016). Ligand Engineering for the Efficient Dye-Sensitized Solar Cells with Ruthenium Sensitizers and Cobalt Electrolytes. Inorg. Chem..

[28] Aghazada S., Gao P., Yella A., Moehl T., Teuscher J., Moser J.-E., Grätzel M., Nazeeruddin M.K. (2016). Unraveling the Dual Character of Sulfur Atoms on Sensitizers in Dye-Sensitized Solar Cells. ACS Appl. Mater. Interfaces.

[29] Aghazada S., Ren Y., Wang P., Nazeeruddin M.K. (2017). Effect of Donor Groups on the Performance of Cyclometalated Ruthenium Sensitizers in Dye-Sensitized Solar Cells. Inorg. Chem..

[30] Medved’ko A.V., Ivanon V.K., Kiskin V.M.A., Sadovnikov A.A., Apostolova E.S., Grinberg V.A., Emets V.V., Chizhov A.O., Nikitin O.M., Magdesieva T.V. (2017). The design and synthesis of thiophene-based ruthenium(II) complexes as promising sensitizers for dye-sensitized solar cells. Dyes Pigment..

[31] Nguyen T.-D., Lin C.-H., Wu C.-G. (2016). Effect of the CF3 Substituents on the Charge-Transfer Kinetics of High-Efficiency Cyclometalated Ruthenium Sensitizers. Inorg. Chem..

[32] Nguyen T.-D., Lan Y.-P., Wu C.-G. (2018). High-Efficiency Cycloruthenated Sensitizers for Dye-Sensitized Solar Cells. Inorg. Chem..

[33] Lavrova M.A., Mishurinskiy S.A., Smirnov D.E., Kalle P., Krivogina E.V., Kozyukhin S.A., Emets V.V., Mariasina S.S., Dolzhenko V.D., Bezzubov S.I. (2020). Cyclometalated Ru(ii) complexes with tunable redox and optical properties for dye-sensitized solar cells. Dalton Trans..

[34] Hu F.-C., Wang S.-W., Chi Y., Robertson N., Hewat T., Hu Y., Liu S.-H., Chou P.-T., Yang P.-F., Lin H.-W. (2014). Geometrical Isomerism of RuII Dye-Sensitized Solar Cell Sensitizers and Effects on Photophysical Properties and Device Performances. Chem. Phys. Chem..

[35] Wu K.-L., Hu Y., Chao C.-T., Yang Y.-W., Hsiao T.-Y., Robertson N., Chi Y. (2014). Dye sensitized solar cells with cobalt and iodine-based electrolyte: The role of thiocyanate-free ruthenium sensitizers. J. Mater. Chem. A.

[36] Colombo A., Dragonetti C., Magni M., Roberto D., Demartin F., Caramori S., Bignozzi C.A. (2014). Efficient Copper Mediators Based on Bulky Asymmetric Phenanthrolines for DSSCs. ACS Appl. Mater. Interfaces.

[37] Takeshita T., Umeda T., Oonishi N., Hara M. (2015). Application of a Noncarboxylated Dye Compound in a Dye-Sensitized Solar Cell Containing a Cyclodextrin Layer. Int. J. Photoenergy.

[38] Park S., Kim H., Jang S., Won J. (2014). Effects of cyclodextrin complexes acting as barriers on TiO2 nanoparticles in DSSCs. J. Photochem. Photobiol. A Chem..

[39] Ou Y., Chen G., Yin J., Yu G.-A., Liu S.H. (2011). Rotaxane based on terpyridyl bimetal ruthenium complexes and β-cyclodextrin as organic sensitizer for dye-sensitized solar cells. J. Coord. Chem..

[40] Swetha T., Niveditha S., Bhanuprakash K., Singh S.P. (2015). Panchromatic Ru (II) Dipyrrins as NCS Free Sensitizers Showing Highest Efficiency for DSSCs. Electrochim. Acta.

[41] Wu K.-L., Huckaba A., Clifford J.N., Yang Y.-W., Yella A., Palomares E., Grätzel M., Chi Y., Nazeeruddin M.K. (2016). Molecularly Engineered Ru(II) Sensitizers Compatible with Cobalt(II/III) Redox Mediators for Dye-Sensitized Solar Cells. Inorg. Chem..

[42] Ngo K.T., Lee N.A., Pinnace S.D., Rochford J. (2017). Engineering of Ruthenium(II) Photosensitizers with Non-Innocent Oxyquinolate and Carboxyamidoquinolate Ligands for Dye-Sensitized Solar Cells. Chem. A Eur. J..

[43] Ashraf S., Akhtar J., Siddiqi H.M., El-Shafei A. (2017). Thiocyanate-free ruthenium(ii) sensitizers with a bi-imidazole ligand in dye-sensitized solar cells (DSSCs). New J. Chem..

[44] Guimaraes R.R., Parussulo A.L., Matias T.A., Toma H.E., Araki K. (2017). Electrostatic blocking barrier as an effective strategy to inhibit electron recombination in DSSCs. Electrochim. Acta.

[45] Subramaniam K., Athanas A.B., Kalaiyar S. (2019). Dual anchored Ruthenium(II) sensitizer containing 4-Nitro-phenylenediamine Schiff base ligand for dye sensitized solar cell application. Inorg. Chem. Commun..

[46] Fiorini V., Marchini E., Averardi M., Giorgini L., Muzzioli S., Dellai A., Argazzi R., Sanson A., Sangiorgi N., Caramori S. (2020). New examples of Ru(ii)-tetrazolato complexes as thiocyanate-free sensitizers for dye-sensitized solar cells. Dalton Trans..

[47] Amthor S., Braun H., Gröne J., Nauroozi D., Jacob T., Rau S. (2019). Tailored protective groups for surface immobilization of ruthenium dyes. Dalton Trans..

[48] Pirashanthan A., Thanihaichelvan M., Mariappan K., Velauthapillai D., Ravirajan P., Shivatharsiny Y. (2021). Synthesis of a carboxylic acid-based ruthenium sensitizer and its applicability towards Dye-Sensitized Solar Cells. Sol. Energy.

[49] Dragonetti C., Colombo A., Magni M., Mussini P.R., Nisic F., Roberto D.M., Ugo R., Valore A., Valsecchi A., Salvatori P. (2013). Thiocyanate-Free Ruthenium(II) Sensitizer with a Pyrid-2-yltetrazolate Ligand for Dye-Sensitized Solar Cells. Inorg. Chem..

